# Genetic Dissection of the Type VI Secretion System in *Acinetobacter* and Identification of a Novel Peptidoglycan Hydrolase, TagX, Required for Its Biogenesis

**DOI:** 10.1128/mBio.01253-16

**Published:** 2016-10-11

**Authors:** Brent S. Weber, Seth W. Hennon, Meredith S. Wright, Nichollas E. Scott, Véronique de Berardinis, Leonard J. Foster, Juan A. Ayala, Mark D. Adams, Mario F. Feldman

**Affiliations:** a Department of Molecular Microbiology, Washington University School of Medicine in St. Louis, St. Louis, Missouri, USA; b Department of Biological Sciences, University of Alberta, Edmonton, Alberta, Canada; c J. Craig Venter Institute, San Diego, California, USA; d Centre for High-Throughput Biology, University of British Columbia, Vancouver, British Columbia, Canada; e CEA, DRT, IG, Genoscope, Évry, France; f CNRS-UMR8030, Évry, France; g Université d’Évry Val d’Essonne, Évry, France; h Centro de Biología Molecular Severo Ochoa (CBMSO), Consejo Superior de Investigaciones Científicas, Universidad Autónoma de Madrid, Madrid, Spain

## Abstract

The type VI secretion system (T6SS) is a widespread secretory apparatus produced by Gram-negative bacteria that has emerged as a potent mediator of antibacterial activity during interbacterial interactions. Most *Acinetobacter* species produce a genetically conserved T6SS, although the expression and functionality of this system vary among different strains. Some pathogenic *Acinetobacter baumannii* strains activate this secretion system via the spontaneous loss of a plasmid carrying T6SS repressors. In this work, we compared the expression of T6SS-related genes via transcriptome sequencing and differential proteomics in cells with and without the plasmid. This approach, together with the mutational analysis of the T6SS clusters, led to the determination of the genetic components required to elaborate a functional T6SS in the nosocomial pathogen *A. baumannii* and the nonpathogen *A. baylyi*. By constructing a comprehensive combination of mutants with changes in the T6SS-associated *vgrG* genes, we delineated their relative contributions to T6SS function. We further determined the importance of two effectors, including an effector-immunity pair, for antibacterial activity. Our genetic analysis led to the identification of an essential membrane-associated structural component named TagX, which we have characterized as a peptidoglycan hydrolase possessing l,d-endopeptidase activity. TagX shows homology to known bacteriophage l,d-endopeptidases and is conserved in the T6SS clusters of several bacterial species. We propose that TagX is the first identified enzyme that fulfills the important role of enabling the transit of T6SS machinery across the peptidoglycan layer of the T6SS-producing bacterium.

## IMPORTANCE

*Acinetobacter baumannii* is one of the most troublesome and least investigated multidrug-resistant bacterial pathogens. We have previously shown that *A. baumannii* employs a T6SS to eliminate competing bacteria. Here we provide a comprehensive analysis of the components of the T6SS of *Acinetobacter*, and our results provide genetic and functional insights into the *Acinetobacter* T6SS. Through this analysis, we identified a novel peptidoglycan hydrolase, TagX, that is required for biogenesis of the T6SS apparatus. This is the first peptidoglycanase specialized in T6SS function identified in any species. We propose that this enzyme is required for the spatially and temporally regulated digestion of peptidoglycan to allow assembly of the T6SS machinery.

## INTRODUCTION

While many species of *Acinetobacter*, a genus of Gram-negative bacteria of the class *Gammaproteobacteria*, are commonly cultivated from environments such as soil and water, several *Acinetobacter* species are important opportunistic pathogens (1). The most clinically relevant member of the genus, *Acinetobacter baumannii*, is rarely isolated outside the hospital setting and is a major health care threat because of extensive drug resistance (2, 3). Environmental and pathogenic strains of *Acinetobacter* species are likely to encounter and interact with other microbes. The factors mediating such interactions are not well known, although cross-kingdom signaling has been shown to affect the outcome of *A. baumannii* interactions with some eukaryotes (4). Furthermore, the ability to withstand desiccation and form biofilms may contribute to *Acinetobacter* species persistence under hostile conditions in their various niches (5–7). The multitude of molecular structures present on the cell surface contribute in a variety of ways to the survival and pathogenicity of these organisms (8).

Secretion systems of Gram-negative bacteria are diverse in terms of both the structural components used to assemble the secretion system and the proteins exported by each apparatus. Among the different secretory systems employed by Gram-negative bacteria, the type VI secretion system (T6SS) has emerged as a widespread mechanism for protein export (9–11). The T6SS delivers effector proteins to both bacterial and eukaryotic cells in a contact-dependent manner, with the exported toxins harboring enzymatic activity deleterious to the target cell (12). There exists a significant diversity in the effector repertoires of T6SS-containing organisms, and delivery depends on the assembly of a core set of 13 conserved T6SS genes (9), called *tss* genes, as well as a variable number of T6SS-associated genes (*tag* genes), that are accessory components that often contribute to regulation. Many T6SS components resemble bacteriophage proteins, leading to models in which the T6SS apparatus mimics an inverted bacteriophage that assembles in the cytoplasm and contracts, eventually releasing its cargo (13). Indeed, structural and functional studies have provided support for this model (14, 15). In addition to secretion of effector proteins, contraction of the T6SS also leads to the export of two hallmark proteins, Hcp and VgrG. Hcp, one of the main components secreted by all functional T6SSs (16), assembles as a hexamer and bears similarity to phage λ tail protein gpV (11, 14, 17). Detection of secreted Hcp in culture supernatants is a well-established molecular marker of a functional T6SS (16). VgrG proteins are similar to the complex formed by gp27/gp5 of T4 phage (17) and can contain effector activity in extended C-terminal domains (18). Although Hcp and VgrG are often codependent for secretion and required for T6SS activity, multiple similar VgrG proteins are usually possessed by a single organism and each may not be essential for Hcp secretion (18–21). Recently, Hcp and VgrG have been shown to be necessary for distinct effector export pathways (22). Hcp can interact with effector substrates via the internal residues of its ring-shaped structure (23), and PAAR domain-containing proteins can interact with the tip of some VgrG trimers (24). Furthermore, genes for effectors are usually located near *vgrG* genes that are essential for the secretion of the effectors (25). These effectors may interact directly with the cognate VgrG or utilize adaptor proteins to facilitate their secretion (25–28). Other important components include the TssB and TssC proteins, which assemble cytoplasmic structures resembling a bacteriophage sheath (29) and change rapidly between extended and contracted states (13). The sheath formed by TssB/TssC, which requires the gp25-like baseplate protein TssE for assembly (13, 30), may physically accommodate the Hcp tubule (31) and is recycled by another essential T6SS component, ClpV (TssH), upon contraction (29). Mutagenesis and functional studies have confirmed the importance of the remaining T6SS components for apparatus function (32, 33), including a membrane complex and other essential structural elements (20, 34–36). Structures spanning the bacterial cell wall, for example, flagella and T3SSs, often require dedicated peptidoglycan (PG)-remodeling enzymes for their assembly (37). Despite the tremendous advances in our understanding of T6SS biogenesis in the past decade, it remains to be determined how this secretory system passes through the PG layer of the T6SS-producing organism.

It has recently come to be appreciated that many *Acinetobacter* species possess an antibacterial T6SS (38–42). However, individual strains vary in the expression of the T6SS, with some exhibiting robust T6SS activity and others possessing an apparently inactive system (38, 41, 42). The T6SS of several *A. baumannii* strains is controlled by plasmid-encoded regulators, and multidrug-resistant strains generally do not show T6SS activity under laboratory conditions (41, 42). Bioinformatic analysis has suggested that the core T6SS components of *Acinetobacter* species are encoded by genes in a single conserved locus, with *vgrG* genes being distributed in various numbers throughout the genome (38, 43, 44). While the *tssM*, *tssB*, and *hcp* genes have been mutated and confirmed as essential for T6SS activity in *Acinetobacter*, the remaining genes in the T6SS cluster, as well as the *vgrG* genes, have not been experimentally tested for their contributions to the T6SS (38, 39, 41).

In this work, we analyzed the genetic requirements for the elaboration of a functional T6SS in *Acinetobacter* species. Furthermore, we evaluated the contributions of VgrG proteins and effectors to apparatus function and antibacterial activity. Finally, we describe a novel structural component of the T6SS, a PG-degrading enzyme we have termed TagX. We found that this enzyme is conserved across several genera and is essential for extracellular export of Hcp. We propose that TagX, by functioning as an l,d-endopeptidase, performs the essential enzymatic step of cell wall degradation, allowing transit of the T6SS tubule.

## RESULTS

### Transcriptomic and genetic analyses reveal novel genes required for the *Acinetobacter* T6SS.

While the proteins required for assembly and function of the T6SS have been investigated in detail in some bacteria, little is known about the requirements for the *Acinetobacter* T6SS. We recently described a novel mechanism of T6SS regulation possessed by several strains of *A. baumannii* (42). In these strains, plasmid-encoded regulators repressed the chromosomally encoded T6SS. Upon spontaneous plasmid loss, and therefore loss of the repressors, the T6SS was activated and functioned as an antibacterial apparatus. However, the precise transcriptional changes that occurred upon plasmid loss were not investigated. We used transcriptome sequencing (RNA-seq) to probe the changes in gene expression between plasmid-containing (T6^−^) and plasmidless (T6^+^) *A. baumannii* ATCC 17978 cells in order to define the components required for a functional T6SS. These transcriptomic data revealed the genes in the previously predicted T6SS cluster that were upregulated in T6^+^ cells (Table 1). The transcriptomic changes were significant, with up to nearly 50-fold upregulation of some genes. Furthermore, distantly located *vgrG* gene clusters also showed significant changes in their transcriptional profile. We then confirmed these changes at the protein level by a whole-cell differential proteomic approach (Table 1). Of the 21 genes identified as statistically significantly differentially expressed by RNA-seq in the main T6SS cluster, we identified and quantified, on the basis of at least two unique peptides, 18 of these proteins, all of which were statistically significantly different between T6^+^ and T6^−^ cells. These data prompted us to genetically dissect the contributions of these proteins to the assembly of the *Acinetobacter* T6SS.

**TABLE 1:** Transcriptomic and differential proteomic analyses of T6SS and VgrG clusters in T6^+^ and T6^−^ *A. baumannii* ATCC 17978

Gene locus	T6SS gene	T6^+^ TPM^a^	T6^−^ TPM^a^	Log_2_-fold difference^b^ (*P* value)^c^	RNA-seq transcript fold difference^b^	Corresponding gene in *A. baylyi* ADP1	TMT-based protein log_2_-fold difference^b^ (*P* value)^c^	No. of peptides identified (Andromeda identification score)
T6SS cluster								
ACX60_11600	*tagX*	37.2 (3.2)	3.4 (1.0)	3.1 (3.3E-28)	8.3	ACIAD2699	NaN^d^	NaN
ACX60_11605	Hypothetical	63.0 (17.0)	2.7 (0.5)	4.0 (2.8E-32)	15.5	ACIAD2698	1.8 (0.00041)	3 (22.204)
ACX60_11610	*tssL*	91.3 (18.4)	4.5 (2.1)	3.9 (6.5E-46)	14.7	ACIAD2697	1.2 (0.028232)	1 (2.4722)
ACX60_11615	*tssK*	259.6 (14.1)	10.8 (1.6)	4.3 (7.8E-158)	19.8	ACIAD2696	1.7 (0.011007)	6 (76.533)
ACX60_11620	*tssA*	221.9 (26.5)	5.2 (0.8)	5.0 (3.8E-134)	32.8	ACIAD2695	2.6 (0.001157)	5 (21.411)
ACX60_11625	*clpV*	258.7 (8.6)	23.1 (2.0)	3.3 (4.8E-144	9.5	ACIAD2694	2.5 (0.002065)	35 (323.31)
ACX60_11630	PAAR	39.2 (15.7)	5.0 (0.9)	2.3 (8.0E-08)	5.1	ACIAD2681	0.9 (0.066403)	1 (12.532)
ACX60_11635	*tagN*	99.4 (9.3)	8.9 (1.5)	3.2 (1.9E-48)	9.0	ACIAD2682	1.5 (0.004644)	6 (54.441)
ACX60_11640	*tagF*	44.4 (12.6)	3.3 (0.8)	3.3 (2.6E-28)	9.9	ACIAD2683	1.7 (0.005088)	4 (22.213)
ACX60_11645	*tssM*	320.3 (16.8)	23.3 (0.8)	3.5 (3.2E-194)	11.7	ACIAD2684	1.8 (0.029757)	35 (323.31)
ACX60_11650	Hypothetical	89.3 (13.9)	4.1 (1.2)	4.1 (6.8E-72)	17.1	ACIAD2685	1.2 (0.000429)	2 (28.15)
ACX60_11655	*tssG*	179.8 (32.2)	4.5 (0.3)	4.9 (3.2E-99)	30.1	ACIAD2686	1.4 (0.000083)	2 (4.6976)
ACX60_11660	*tssF*	134.9 (28.3)	4.5 (0.5)	4.6 (1.1E-103)	23.5	ACIAD2687	2.7 (0.018475)	4 (23.9)
ACX60_11665	*tssE*	1078.5 (165.7)	30.2 (2.3)	4.8 (3.2E-167)	28.7	ACIAD2688	NaN	NaN
ACX60_11670	*hcp*	11801.2 (588.2)	198.8 (18.9)	5.6 (0)	49.4	ACIAD2689	2.8 (0.000359)	11 (139.41)
ACX60_11675	*tssC*	1823.6 (258.4)	30.5 (1.6)	5.6 (0)	49.0	ACIAD2690	2.7 (0.000598)	23 (323.31)
ACX60_11680	*tssB*	1516.7 (145.5)	34.7 (7.8)	5.1 (3.3E-206)	35.2	ACIAD2691	3.1 (0.000546)	14 (253.32)
ACX60_11685	Hypothetical	545.2 (6.2)	9.3 (0.6)	5.5 (8.2E-202)	46.3	ACIAD2693	2.7 (0.001271)	9 (235.49)
ACX60_11690	Hypothetical	600.3 (50.0)	125.4 (19.9)	2.0 (8.7E-43)	4.0	ACIAD3112/3113	1.1 (0.002987)	18 (321.94)
ACX60_11695	*tse3* effector	167.0 (37.0)	14.6 (0.2)	3.2 (2.6E-77)	9.5	ACIAD3114	NaN	NaN
ACX60_11700	*vgrG3*	96.3 (10.7)	47.8 (1.4)	0.8 (1.2E-08)	1.8	ACIAD3115	1.2 (0.013778)	13 (1.2162)
ACX60_11705	Hypothetical	6.0 (2.3)	2.7 (0.6)	0.7 (3.0E-01)	1.6	None	0.1 (0.646629)	1 (1.8381)
VgrG1 cluster								
ACX60_17640	Hypothetical	16.2 (3.2)	3.4 (2.3)	1.7 (2.8E-4)	3.2	None	NaN	NaN
ACX60_17645	Hypothetical	9.6 (40)	0.4 (0.4)	2.6 (7.9E-6)	6.0	None	NaN	NaN
ACX60_17650	Hypothetical	34.8 (12.5)	1.5 (1.0)	3.5 (4.8E-15)	11.6	None	NaN	NaN
ACX60_17655	Hypothetical	37.6 (5.3)	2.0 (1.2)	3.5 (3.8E-19)	11.1	None	1.7 (0.010153)	7 (67.368)
ACX60_17660	*tse1*	328.8 (53.3)	12.0 (1.7)	4.4 (2.0E-117)	21.6	None	2.3 (0.003947)	1 (1.6312)
ACX60_17665	*vgrG1*	203.4 (27.4)	36.5 (1.0)	2.2 (9.11E-59)	4.8	Multiple *vgrG* genes	2.4 (0.011138)	16 (222.15)
ACX60_17670	Hypothetical	6.7 (2.9)	1.4 (0.3)	1.6 (2.7E-3)	3.0	None	NaN	NaN
VgrG2 cluster								
ACX60_15360	*tsi2* (*tdi*)	192.3 (17.0)	103.2 (2.9)	0.7 (2.3E-7)	1.6	None	0.1 (0.350265)	9 (114.72)
ACX60_15365	*tse2* (*tde*)	158.9 (17.2)	64.0 (3.3)	1.1 (1.8E-15)	2.1	None	0.4 (0.135599)	8 (65.825)
ACX60_15370	*vgrG2*	143.1 (15.3)	200.4 (13.8)	−0.7 (2.7E-7)	−1.6	Multiple *vgrG* genes	1.0 (0.049891)	12 (33.815)
VgrG4 cluster								
ACX60_00585	*vgrG4*	65.5 (9.4)	23.2 (2.6)	1.3	2.4	Multiple *vgrG* genes	NaN	NaN
ACX60_00590	Hypothetical	1.3 (0.2)	3.9 (0.1)	−0.7	−1.6	None	NaN	NaN
ACX60_00595	Hypothetical	1.8 (1.8)	6.2 (4.1)	−0.3	−1.2	None	NaN	NaN
ACX60_00600	Hypothetical	0.4 (0.7)	0.0 (0.0)	0.2	1.1	None	NaN	NaN
ACX60_00605	*tse4* (*tae*)	13.0 (1.6)	8.2 (2.1)	0.5	1.4	None	NaN	NaN
ACX60_00610	*tsi4*	19.2 (8.6)	8.8 (3.1)	0.8	1.8	None	0.2 (0.283492)	10 (105.54)
ACX60_00615	Hypothetical	5.4 (0.7)	3.2 (1.4)	0.5	1.4	None	NaN	NaN

a Data shown are mean values (standard deviations) of three biological replicate samples.

b Between T6+ and T6−.

c Statistical significance was tested by using DESEq2.

d NaN, not detected.

**FIG 1:**
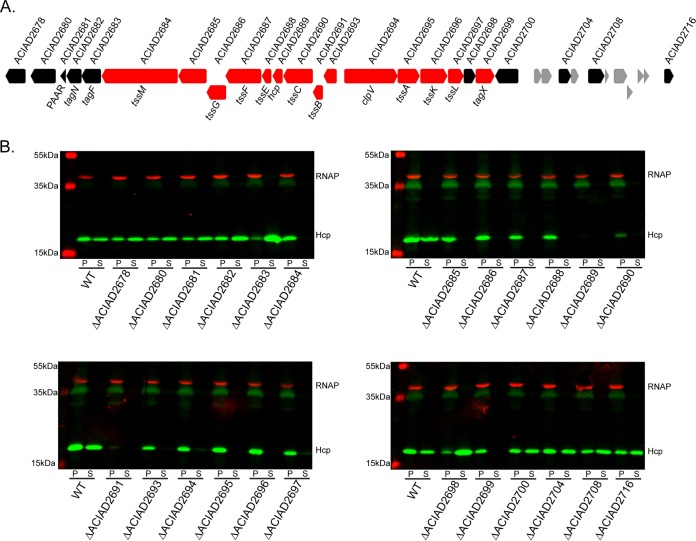
(A) Genetic organization of the T6SS locus in *A. baylyi* ADP1. Locus numbers are indicated at the top, and relevant T6SS nomenclature is shown at the bottom. Genes in red are essential for Hcp secretion, and those in black are not essential or act to enhance secretion. Genes in gray were not tested. (B) Western blot assays probing for Hcp expression and secretion in whole-cell pellets (P) or supernatants (S) of the *A. baylyi* ADP1 strains indicated. Numbers represent the ACIAD gene numbers. RNA polymerase (RNAP) was used as a loading and lysis control. WT, wild type.

Recently, a complete collection of null mutants of nonpathogenic *A. baylyi* ADP1 was constructed that included genes that encode proteins expected to be involved in T6SS apparatus assembly and activity (43). *A. baylyi* ADP1 possesses a T6SS that secretes the conserved Hcp protein under standard laboratory growth conditions (38), and it was previously demonstrated that this secretion system contributes to antibacterial activity against *Escherichia coli* (40). Furthermore, the genes in this cluster are highly conserved across all T6SS-producing *Acinetobacter* species (38). Using our transcriptomic data as a guide, we tested several mutants with changes within and surrounding the predicted T6SS cluster for ADP1 T6SS function (Fig. 1A). Hcp secretion was abolished in 14 of the mutants with changes that clustered in a 15-gene tract from ACIAD2684 to ACIAD2699 (Fig. 1B), and this confirms that this cluster comprises the core T6SS genes in this organism. The *tagN* (ACIAD2682), *tagF* (ACIAD2683), and ACIAD2698 mutants secreted Hcp to a greater extent than the wild type did. While ACIAD2698 has no homologs outside the genus *Acinetobacter*, TagN contains a PG binding domain and is present in *Burkholderia* T6SS-1 (45) and TagF is a repressor of the *Pseudomonas aeruginosa* H1 T6SS (46).

Excluding the *hcp* mutant itself, most of the mutant strains expressed levels of Hcp comparable to those of wild-type ADP1 when analyzed by Western blotting, indicating that a lack of expression was not a factor in the loss of Hcp secretion (Fig. 1B). However, *tssB* and *tssC* mutants expressed a detectably lower level of Hcp, which could account for the loss of Hcp secretion. As *tssB* and *tssC* are located upstream of *hcp*, it is possible that mutation of these genes affected the downstream expression of *hcp*. However, the *tssB* and *tssC* genes are both essential for Hcp secretion in other bacteria (32). ACIAD2685, ACIAD2693, and ACIAD2699 do not have characterized homologs in other T6SS-producing organisms, indicating that they represent novel genetic components of the *Acinetobacter* T6SS. ACIAD2685 is predicted to encode a membrane protein, and ACIAD2693 is predicted to encode a signal peptide and therefore probably functions in the periplasm. The protein encoded by ACIAD2699 is predicted to contain a C-terminal peptidase domain (pfam13539) similar to the PG-cleaving VanY peptidase superfamily of proteins but does not resemble any known T6SS components.

These results establish the core components of the T6SS of *A. baylyi* ADP1 and, considering their conserved nature in this genus (38), offer insight into necessary T6SS components in other *Acinetobacter* species.

### Establishing the contribution of VgrG proteins to apparatus formation and bacterial killing identifies important antibacterial effectors.

**FIG 2:**
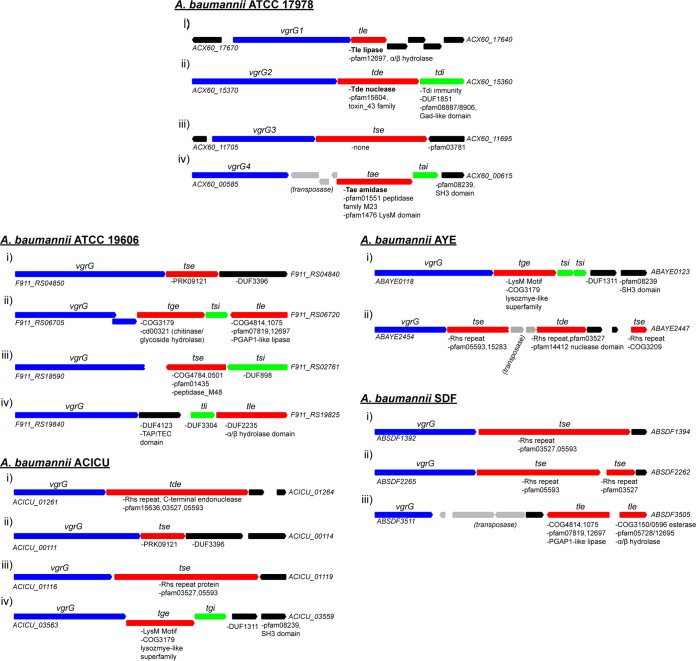
Schematic of the *vgrG* clusters present in selected *A. baumannii* strains. The *vgrG* genes are blue, the putative *tse* effectors are red, and the *tsi* immunity genes are in green. Locus tags are shown for the first and last genes of each cluster, and conserved domains are indicated below the genes where relevant.

VgrG proteins are secreted T6SS components that can be essential for forming a functional T6SS but also play a role in the secretion of other components (10, 24, 25). These proteins can mediate the secretion of effectors encoded downstream or have effector functions in extended C-terminal domains (16, 18, 25). The number of *vgrG* genes possessed by *Acinetobacter* species varies but is generally between two and four (Fig. 2) (38). *A. baumannii* ATCC 17978 contains four VgrG proteins (VgrG1 to -4), none of which contains an identifiable effector domain. However, near each *vgrG* gene is a gene for a putative effector, suggesting that these VgrG proteins act as adaptors for the secretion of cognate effectors (Fig. 2). Indeed, all of the VgrG proteins in *A. baumannii* ATCC 17978 contain a C-terminal DUF2345 domain that has been shown to play a role in stabilizing VgrG-effector interactions (28). Our bioinformatic analysis indicated that *A. baumannii* possesses a diverse repertoire of effectors encoded by *vgrG* clusters, with some seemingly susceptible to transposase integration (e.g., strain AYE) (Fig. 2). The predicted effector downstream of *A. baumannii* ATCC 17978 *vgrG1* contains domains associated with lipases, and several type VI lipase effectors (Tle) have been previously shown to target lipidic substrates accessible from the periplasm, with cognate immunity proteins located in the periplasm (47). Furthermore, this putative effector contains a GXSXG motif typical of some Tle family members (47). This suggests that the enzyme encoded by a gene downstream of *vgrG1* is a Tle, and immunity would be due to a protein encoded by a nearby gene. Immunity proteins are typically more difficult to predict *in silico*; however, the protein of unknown function encoded by ACX60_17670 contains a signal peptide directing it to the periplasm, which is the expected site of Tle activity (Fig. 2). The predicted effector encoded by a gene near *vgrG2* contains a nuclease domain, and the downstream immunity protein contains a GAD-like domain and a DUF1851 domain. A similar nuclease (type VI DNase effector, Tde) and immunity pair were found in *Agrobacterium tumefaciens*, with the Tde effector being delivered in a T6SS-dependent manner (48). Indeed, those authors bioinformatically identified this *A. baumannii* effector as a member of the Tde superfamily containing the requisite HXXD catalytic motif. The predicted effector protein in the *vgrG3* cluster did not show homology to any known effector domains, but our experimental data suggest that it plays an important role in bacterial killing. While T6SS effectors are generally named on the basis of their characterized biochemical activity, in the absence of such data, we have tentatively named this gene *tse* (type six effector). The effector downstream of *vgrG4* contains a LysM PG binding motif and an M23 peptidase family domain, indicating that it probably targets the PG, which is a common target for T6SS Tae effectors (49). Furthermore, the predicted immunity protein contains a signal peptide, which indicates that it interacts with an effector targeted to the periplasm.

By our RNA-seq and differential proteomic analyses, we found that most of the *vgrG* genes and their associated cluster were upregulated in T6^+^ *A. baumannii* (Table 1). The *vgrG3* gene, which is located upstream of the main T6SS cluster, was upregulated 1.8-fold, while *tse* transcripts increased 9.5-fold. Similarly, transcripts of *vgrG1* and its associate *tle* increased 4.8- and 21.6-fold, respectively. Interestingly, *vgrG2* expression decreased by 1.6-fold, while the effector and immunity genes were upregulated 2.1- and 1.6-fold, respectively. However, expression of this cluster was robust in both T6^+^ and T6^−^ cells, as assessed by counts of transcripts per million (TPM), which suggests that the cluster containing *vgrG2* is regulated in a different manner. We found a significant increase in the transcription of *vgrG4* (upregulated 2.4-fold) in T6^+^ cells; however, the changes in the expression of the predicted downstream effector and immunity proteins encoded by *tae* and *tai* (increased 1.4- and 1.8-fold, respectively) did not reach statistical significance. Collectively, these data support the notion that many of these *vgrG* clusters are coregulated with the core T6SS in this organism.

**FIG 3:**
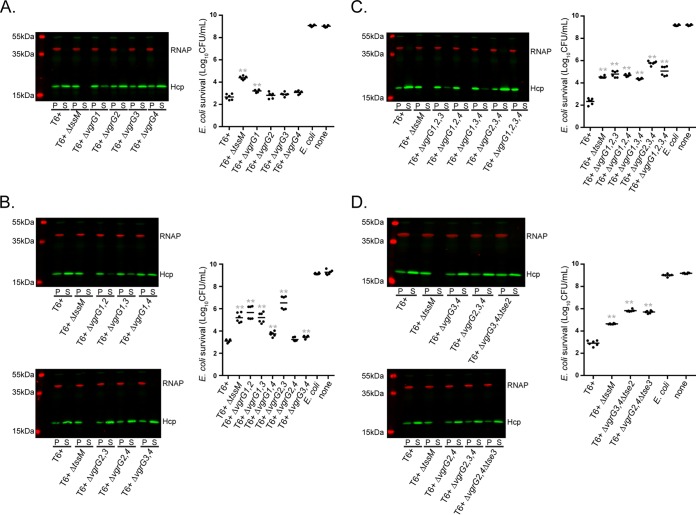
Hcp secretion and bacterial killing by T6^+^ *A. baumannii* ATCC 17978 *vgrG* mutants. Western blot assays probing for Hcp and RNA polymerase (RNAP) and *E. coli* killing assays were performed with single *vgrG* deletion mutants (A), double *vgrG* deletion mutants (B), triple and quadruple *vgrG* deletion mutants (C), and *tse2* and *tse3* effector mutants (D) in the Δ*vgrG34* or Δ*vgrG24* background, respectively. For the bacterial killing assay graphs, the *A. baumannii* predator strains indicated are on the *x* axis and the log-transformed surviving *E. coli* MG1655R CFU count is on the *y* axis. A drug-sensitive strain of *E. coli* or no predator (none) was used as a control. The results shown are from two independent experiments performed in triplicate. Asterisks indicate statistical significance compared to wild-type T6^+^ (unpaired, two-tailed Student *t* test; **, *P* < 0.01).

With the core components of the T6SS delineated, we sought to determine the roles of these VgrG proteins in *A. baumannii* T6SS function. Using a recently described mutagenesis strategy (50), we generated a complete series of mutants lacking one, two, three, or all four *vgrG* genes in every possible combination in the *A. baumannii* ATCC 17978 T6^+^ background. These strains were then assessed for the ability to express and secrete Hcp, as well as the ability to kill *E. coli* prey cells (Fig. 3A to D). As a control for Hcp secretion and *E. coli* killing, we used a mutant with a change in the essential *tssM* gene that prevents apparatus assembly and therefore T6SS-mediated bacterial killing (38).

The results of our Hcp secretion and bacterial killing experiments with the full suite of *vgrG* mutants are summarized in Table 2. On the basis of our data, *vgrG1* can be considered the most significant contributor to Hcp secretion in T6^+^ *A. baumannii*. When *vgrG1* was mutated, secretion of Hcp was impaired; in all other single *vgrG* mutants, Hcp was secreted to wild-type levels (Fig. 3A). Double *vgrG* mutants in which *vgrG1* was deleted secreted markedly less Hcp than the wild type, although there were subtle differences among the Δ*vgrG12*, Δ*vgrG13*, and Δ*vgrG14* mutants (Fig. 3B). The remaining double mutants, all of which contained a functional copy of *vgrG1*, did not show any Hcp secretion defect. Minimal amounts of Hcp were detectable in the supernatants from the Δ*vgrG123* and Δ*vgrG134* triple mutants, which suggests that VgrG4 or VgrG2 is required for the low levels of Hcp secretion observed in the absence of VgrG1 (Fig. 3C). A total lack of Hcp in supernatants from the Δ*vgrG124* mutant indicated that VgrG3 does not play a role in Hcp secretion. Interestingly, Hcp secretion was identical to that of wild-type bacteria in the Δ*vgrG234* strain, which solely contains VgrG1. Expression of VgrG1 from a plasmid in either the Δ*vgrG1* or the Δ*vgrG124* background reconstituted Hcp secretion, confirming the essential role of VgrG1 in Hcp export (see Fig. S1 in the supplemental material). As expected, no Hcp was secreted by the strain lacking all four *vgrG* genes (Fig. 3C).

**TABLE 2:** Summary of Hcp secretion and bacterial killing data in Fig. 3^a^

*A. baumannii* ATCC 17978 strain	Hcp secretion^a^	Bacterial killing^b^
T6^+^	++++	+++
T6^+^ Δ*tssM*	−	+
T6^+^ *ΔvgrG1*	++	++
T6^+^ Δ*vgrG2*	++++	+++
T6^+^ Δ*vgrG3*	++++	+++
T6^+^ Δ*vgrG4*	++++	+++
T6^+^ Δ*vgrG12*	+	+
T6^+^ Δ*vgrG13*	++	+
T6^+^ Δ*vgrG14*	+++	++
T6^+^ Δ*vgrG23*	++++	+
T6^+^ Δ*vgrG24*	++++	+++
T6^+^ Δ*vgrG34*	++++	++
T6^+^ Δ*vgrG123*	+	+
T6^+^ Δ*vgrG124*	−	+
T6^+^ Δ*vgrG134*	+	+
T6^+^ Δ*vgrG234*	++++	+
T6^+^ Δ*vgrG1234*	−	+
T6^+^ Δ*vgrG34* Δ*tse2*	++++	+
T6^+^ Δ*vgrG24* Δ*tse3*	++++	+

a Symbols: ++++, wild type; +++, moderately impaired; ++, impaired; +, severely impaired; −, not detected.

b Symbols: +++, wild type; ++, impaired; +, severely impaired.

Assessment of antibacterial activity in the various *vgrG* mutant strains revealed that deletion of *vgrG1* had a small but significant effect on *E. coli* survival, while the remaining single *vgrG* mutants had no appreciable bacterial killing defect compared to the wild type (Fig. 3A). This result is consistent with the Hcp secretion defect in the Δ*vgrG1* mutant strain. Many of the double *vgrG* mutants were severely impaired in the ability to kill *E. coli*. The Δ*vgrG12* and Δ*vgrG13* mutants were attenuated to the level of the Δ*tssM* strain, while the Δ*vgrG14* mutant had a less pronounced yet still significant defect (Fig. 3B). This result mirrors the corresponding Hcp secretion phenotypes. Of the remaining double mutants, each of which retained wild-type levels of Hcp secretion, the Δ*vgrG23* mutant was particularly defective in bacterial killing. In fact, *E. coli* survived to a greater extent when incubated with this mutant than when incubated with the Δ*tssM* strain. We found a small but statistically significant loss of bacterial killing by the Δ*vgrG34* strain, whereas no phenotypic difference was detected between Δ*vgrG24* mutant and wild-type bacteria. All triple mutants, regardless of the combination, were unable to kill *E. coli* in a T6SS-dependent manner (Fig. 3C). Of particular note is the loss of bacterial killing by the Δ*vgrG234* mutant strain, which was the only triple mutant able to secrete Hcp to wild-type levels, suggesting that any effectors requiring VgrG1 for export are not sufficient to kill *E. coli*. Predictably, mutation of all four *vgrG* genes abrogated bacterial killing (Fig. 3C).

The above-described experiments showed that Δ*vgrG24* and Δ*vgrG34* mutant strains retained wild-type (or nearly wild-type) levels of Hcp secretion and antibacterial activity, while the Δ*vgrG234* strain secreted Hcp to similar levels but was unable to kill *E. coli*. This suggested that the putative effectors encoded by genes near *vgrG2* and *vgrG3*, Tde and Tse, respectively, may play a key role in bacterial killing. We therefore constructed Δ*vgrG34* Δ*tde* and Δ*vgrG24* Δ*tse* mutants and assessed the abilities of these strains to secrete Hcp and kill *E. coli*. While these mutants secreted wild-type levels of Hcp, neither was able to kill *E. coli*, indicating that Tse2 and Tse3 are important mediators of antibacterial activity (Fig. 3D). Furthermore, expression of Tdi in *E. coli* was protective against killing by the Δ*vgrG34* strain, confirming Tde and Tdi as an effector-immunity pair (see Fig. S2 in the supplemental material).

### Identification of a PG-hydrolyzing enzyme essential for T6SS function.

**FIG 4:**
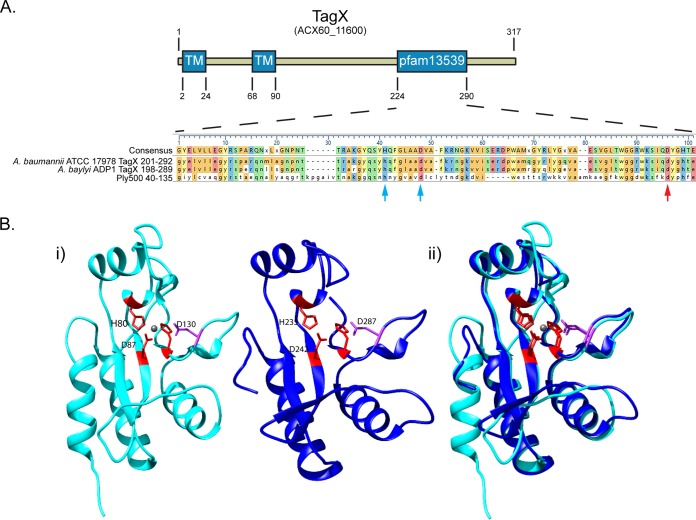
The TagX protein of *A. baumannii* ATCC 17978*.* (A) At the top is a schematic of TagX showing the locations of the predicted transmembrane (TM) domains and the pfam13539 peptidase domain, which resembles members of the VanY superfamily. At the bottom is an alignment of the *A. baumannii* and *A. baylyi* TagX pfam13539 domains with the *Listeria* phage endolysin PlyA500 (accession no. AAY52812). Relevant metal-coordinating residues are indicated by the blue arrows, and the red arrow shows the general base in the active site of the characterized Ply500 enzyme. (B, i) The crystal structure of the enzymatically active domain Ply500 (Protein Data Bank code 2VO9) (53, 54) (left) and the modeled structure of the C-terminal end of *A. baumannii* TagX. The metal-coordinating residues are red, and the general base residue is purple. (B, ii) Overlay of the crystal structure of Ply500 and the modeled structure of TagX. Modeling was performed with SWISS-MODEL (80).

**FIG 5:**
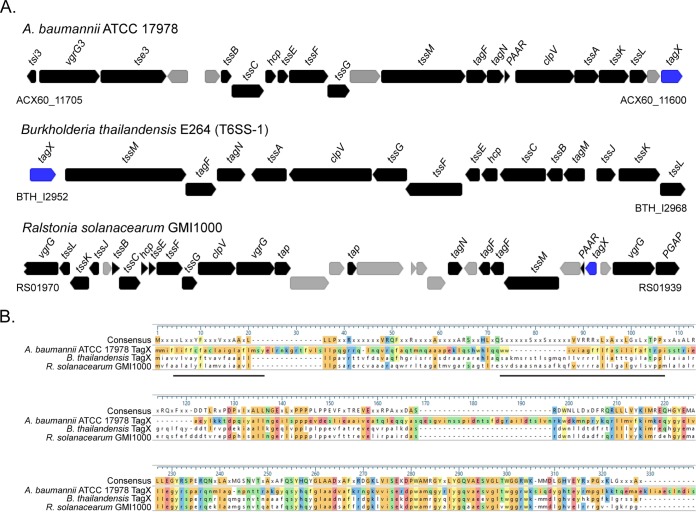
(A) Schematic of the core T6SS clusters from *A. baumannii* ATCC 17978, *B. thailandensis* E264, and *R. solanacearum* GMI1000. Black arrows represent T6SS-related genes, and *tagX* is blue. Genes with unknown functions are gray. (B) Alignment of the full-length TagX proteins from these three bacteria with the predicted transmembrane residues of the *A. baumannii* TagX underlined.

While much progress has been made in understanding the biogenesis and formation of a functional T6SS, it remains unknown how the assembled apparatus transits the PG layer of the T6SS-producing organism (51). In our analysis of the *A. baylyi* ADP1 T6SS, we identified ACIAD2699 as essential for Hcp secretion. The homolog of this gene in *A. baumannii* ATCC 17978 (ACX60_11600) was coregulated with the T6SS (Table 1). This gene encodes a protein with two predicted transmembrane domains and a C-terminal peptidase domain found in the PG-cleaving VanY peptidase superfamily of proteins (pfam13539) but does not resemble any characterized T6SS component from other bacteria (Fig. 4A; see Fig. S3 in the supplemental material). Analysis of protein charge distribution revealed a stretch of positive charges directly after the first predicted transmembrane domain and, on the basis of the “positive-inside rule,” would position the C-terminal enzymatic domain in the periplasm (see Fig. S3) (52). Bioinformatic searches, alignments, and *in silico* modeling showed that this C-terminal domain shared metal-coordinating and active-site residues with a *Listeria* phage endolysin, Ply500, an l-alanoyl-d-glutamate endopeptidase that was previously characterized and crystallized (53, 54) (Fig. 4A and B). Furthermore, this gene is conserved in the T6SS clusters of all *Acinetobacter* species and a homolog is also present in the T6SS clusters of *Burkholderia thailandensis* and *Ralstonia solanacearum* (Fig. 5A and B). We reasoned that this enzyme, which we have termed TagX (type VI-associated gene X), in keeping with previous nomenclature, could play an essential role in T6SS tubule transit through the PG layer of T6SS-producing *Acinetobacter* bacteria.

**FIG 6:**
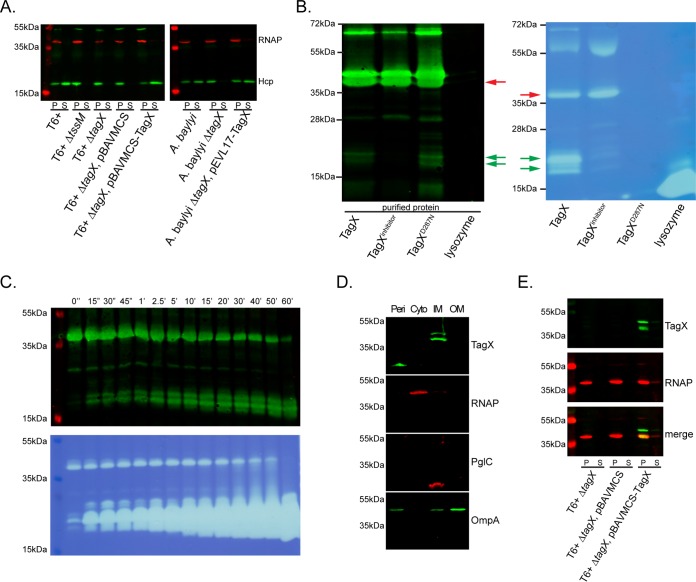
TagX is a membrane-anchored PG hydrolase. (A) Expression and secretion of Hcp and RNA polymerase (RNAP) in pellet (P) and supernatant (S) samples of wild-type *A. baumannii* (T6^+^), the Δ*tssM* mutant, the Δ*tagX* mutant, the Δ*tagX* mutant vector control, and the complemented Δ*tagX* mutant. (B) Expression and secretion of Hcp in P and S samples of wild-type *A. baylyi*, the Δ*tagX* mutant, and the complemented mutant strain. (B, left) Western blot analysis of purified TagX, TagX plus a protease inhibitor (TagX^inhibitor^), and the TagX^D287N^ mutant protein. Proteins were detected with an anti-His polyclonal antibody. (B, right) Zymogram assay with purified TagX proteins and lysozyme as a control. Red and green arrows indicate areas of PG degradation and the corresponding bands in the Western blot. (C) Limited proteolysis of purified TagX. TagX was treated with proteinase K for various times (0 to 60 min), run on an SDS-PAGE gel, and analyzed by Western blotting (top) or loaded onto a gel impregnated with PG for zymogram analysis (bottom). (D) Cellular fractionation studies of TagX in periplasmic (Peri), cytoplasmic (Cyto), inner membrane (IM), and outer membrane (OM) fractions of *A. baumannii* with RNAP, PglC, and OmpA as localization controls. (E) P and S samples of the T6^+^ *A. baumannii* tagX mutant and the *tagX* mutant containing the control vector or expressing TagX were analyzed by Western blotting for TagX expression. Low levels of TagX in the supernatant correspond to leakage of RNAP, indicating that some lysis occurs.

We mutated the *tagX* homolog in *A. baumannii* ATCC 17978 and confirmed that this strain was unable to secrete Hcp (Fig. 6). Importantly, Hcp secretion was restored in *A. baumannii* and *A. baylyi* tagX mutants upon expression of the respective TagX protein from a plasmid (Fig. 6). Using the alignment against the Ply500 PG hydrolase as a guide, we identified conserved amino acids comprising the putative active-site and metal-coordinating residues of TagX. We reasoned that if TagX were a PG hydrolase, mutation of these sites would abrogate PG hydrolysis by this enzyme. We cloned, expressed, and purified C-terminally His-tagged full-length TagX and a TagX mutant protein with a change in the putative active site (TagX^D287N^) from *E. coli* (Fig. 6B; see Fig. S4 in the supplemental material). Expression and purification of the wild-type and TagX^D287N^ proteins were similar. Western blot analysis of purified TagX showed that the protein ran as two predominant bands at a molecular mass of ~40 kDa, near the predicted molecular mass of 36.2 kDa, and also contained a species with a higher molecular mass of ~80 kDa, possibly a dimer. Several degradation products that maintained anti-His reactivity were observed when the protein was purified from *E. coli* without protease inhibitors (to prevent inhibition of TagX enzymatic activity), indicating that they contained the predicted catalytic domains (Fig. 6B). Addition of protease inhibitors lacking EDTA prevented this degradation (Fig. 6B).

To assess the ability of TagX to cleave PG, we performed PG zymogram assays with gels impregnated with PG isolated from *A. baumannii*. After the PG zymograms were renatured and stained, only lanes containing TagX, but not TagX^D287N^, resulted in clear bands of PG hydrolysis (Fig. 6B). Interestingly, of the two predominant protein bands detected by Western blotting, only the lower-molecular-mass species showed activity, which suggests that the full-length enzyme is inactive. Further, despite being present in small quantities, TagX degradation products were fully active, degrading PG comparably to the higher-molecular-mass species. We further assessed the stability and activity of TagX by limited proteolysis with proteinase K (55). In these time course experiments, the full-length enzyme was almost fully degraded by proteinase K treatment (Fig. 6C, top). In contrast, the C-terminal fragments were completely resistant to protease activity and accumulated throughout the experiment, presumably from cleaved full-length TagX. The accumulation of the protease-resistant C-terminal fragment correlated with a very significant increase in PG hydrolysis by these samples, as shown by zymogram assay (Fig. 6C, bottom). At 60 min, the C-terminal fragment was almost exclusively present. The protein levels of full-length TagX at time zero and the C-terminal fragment at 60 min were not markedly different, yet the C-terminal fragment cleaved PG to a much greater extent than full-length TagX at the respective time points. This suggests that the C-terminal fragment displays higher enzymatic activity than the full-length protein and, considering its resistance to protease digestion, suggests a possible regulatory mechanism.

We performed protein localization studies by fractioning *A. baumannii* cells expressing TagX. As predicted, TagX localized to the inner membrane fraction (Fig. 6D) but was not detected in the cytoplasm or outer membrane fraction. A His-tagged C-terminal degradation product of TagX was detected in the periplasmic fraction, which supports the prediction that the pfam13539 domain is oriented toward the periplasm. The localization of RNA polymerase (cytoplasm), PglC (inner membrane) (56), and OmpA (outer membrane) (57) was determined as a control for the isolation of each fraction. Although OmpA was observed in three fractions, it was enriched in the outer membrane, as expected. Additional experiments revealed that TagX was not secreted into culture supernatants (Fig. 6E), indicating that TagX is a bona fide structural element of the T6SS.

**FIG 7:**
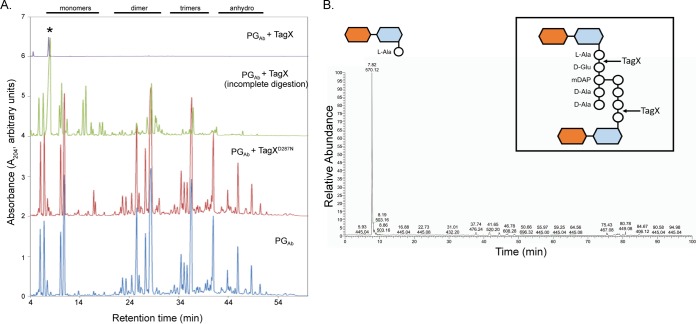
TagX is an l,d-endopeptidase. (A) Chromatographic analysis by HPLC of isolated *A. baumannii* PG (PG_Ab_). PG was digested with TagX, incompletely digested with TagX, digested with TagX^D287N^, or left without enzyme addition. After treatment, the PG was subsequently digested with muramidase and the resulting muropeptides were analyzed by HPLC. Peaks corresponding to PG monomers, dimers, trimers, and anhydromuropeptides are indicated at the top. The asterisk indicates the peak in the TagX-digested PG_Ab_ sample that was further selected for MS characterization. (B) MS analysis of the peak selected from the HPLC trace. The major peak at *m*/*z* 570 corresponds to a PG monomer containing l-alanine. The inset shows the proposed TagX cleavage site on a PG dimer. mDAP, *meso*-diaminopimelic acid.

In order to determine its site of cleavage, we incubated purified TagX and TagX^D287N^ with PG from *A. baumannii* and performed high-performance liquid chromatography (HPLC) and mass spectrometry (MS) to assess cleavage products. PG incubated with TagX^D287N^ or buffer alone looked similar by HPLC (Fig. 7A). Conversely, PG partially digested with TagX showed evidence of degradation, with a large peak of a monomeric species eluting at ~7 min. Upon complete digestion, this was the only peak remaining. MS analysis of this peak showed that it was composed of the PG fragment *N*-acetylglucosamine–*N*-acetylmuramic acid attached to l-Ala, which is the first amino acid residue of the peptide stem (Fig. 7B). These results indicate that the cleavage site of TagX, which enzymatically acts as an l,d-endopeptidase, is between the l-Ala and d-Glu residues of the PG pentapeptide (Fig. 7B, inset).

## DISCUSSION

Great strides have been made in our understanding of the function, biogenesis, and regulation of the bacterial T6SS since its formal description a decade ago (10, 11). However, recent studies have also made it clear that the T6SS can display variations among different species and even among strains of a species or cells of a population. Functionally, the T6SS can be used as an antibacterial, antieukaryotic, or both types of weapon, depending on the organism(s) (58). While many T6SS components are conserved across bacteria, others are species specific. Finally, a diverse range of strategies have evolved to regulate T6SS activity among different organisms (51, 59). For these reasons, it is important to experimentally study the T6SS of the organism of choice in order to delineate the distinctive aspects of this secretory apparatus that may differ from those of other bacteria. Our results further emphasize this point, as we have shown that the T6SS of *Acinetobacter* species, while encoding many components already shown to be important for T6SS function in other bacteria, contains several novel genes that are essential for Hcp secretion but are not included among those that encode other characterized T6SSs. Our analysis showed that 14 genes, *ACIAD2684* to *ACIAD2697* and *ACIAD2699*, are essential for Hcp secretion in *A. baylyi*. The systematic mutagenesis of the T6SS of *Edwardsiella tarda*, *Agrobacterium tumefaciens*, and *Vibrio cholerae* has yielded important insights into the core genes required for T6SS activity (32, 33, 60). In general, our results are consistent with those of previous studies of other bacterial species. This includes a PAAR protein (ACIAD2681) that, when mutated by itself, has no effect on Hcp secretion but, if mutated in combination with two other PAAR proteins encoded by genes located elsewhere on the chromosome, blocks T6SS (24). Most of the *A. baylyi* genes required for Hcp secretion, and thus T6SS activity, are also required for T6SS in *E. tarda*, *V. cholerae*, and *A. tumefaciens*. However, our analysis has uncovered three essential T6SS genes (ACIAD2685, ACIAD2693, and ACIAD2699) that, to our knowledge, have not previously been implicated in T6SS activity and are conserved across *Acinetobacter* species. This indicates that these proteins play an important role in T6SS biogenesis in this genus, but their precise roles remain to be determined. *Acinetobacter* species do not produce a readily identifiable homolog of TssJ (38), a lipoprotein required for T6SS activity in some organisms, and thus, these proteins may compensate for this. Mutation of the gene for ACIAD2698 increased Hcp secretion, indicating that it may perform some regulatory function. Finally, we have identified a novel role for ACIAD2699 (*tagX*), as discussed below, which highlights the importance of examining individual T6SS clusters.

VgrG proteins are secreted in a T6SS-dependent manner and can either contain toxic domains or facilitate the secretion of other effectors (18, 25, 26). These proteins can also be essential for Hcp secretion and thus T6SS function. In *V. cholerae*, mutation of *vgrG1* or *vgrG2*, but not *vgrG3*, prevents Hcp secretion (18), although *vgrG3* mutation results in a detectable Hcp secretion defect (61). Similarly, in *P. aeruginosa*, *vgrG1a* and *vgrG1c* are required for efficient Hcp secretion and Hcp was undetectable in the double mutant (19). A third *vgrG*, *vgrG1b*, was dispensable for the secretion of Hcp (19). In addition to their importance in apparatus assembly, it has now become apparent that many VgrG proteins facilitate the secretion of cognate effectors (25, 26). Our RNA-seq, differential proteomic, and bioinformatic analyses identified the putative *vgrG* clusters in several strains of *A. baumannii*, and although the VgrG proteins were themselves not predicted to contain effector domains, all were encoded by genes near genes for predicted effectors with various enzymatic functions. In *A. baumannii* ATCC 17978, we identified the presence of four *vgrG* genes that were scattered throughout the genome. Their proximity to proteins with predicted toxin domains suggested that these VgrG proteins may facilitate effector secretion. While we did not assess the secretion of these proteins in this study, our mutational strategy showed that VgrG1 is necessary for efficient Hcp secretion, similar to what was seen in *P. aeruginosa*, and deletion of *vgrG1* resulted in a modest bacterial killing defect. Furthermore, the sole presence of VgrG1 was sufficient for wild-type levels of Hcp secretion in the Δ*vgrG234* mutant, confirming its importance for Hcp export. Because mutation of *vgrG1* affected T6SS apparatus formation, mutants lacking this gene in combination with other *vgrG* genes are difficult to assess for their roles in bacterial killing. However, our finding that several double mutants (which contained a functional *vgrG1* gene) lost the ability to kill *E. coli* provides important insights into the roles of these other *vgrG* genes in antibacterial activity. For example, the Δ*vgrG23* mutant secreted wild-type levels of Hcp but did not kill *E. coli*, which suggests that the presence of VgrG1 and VgrG4 is not sufficient to kill *E. coli*. One hypothesis for this is that the effectors downstream of VgrG1 and VgrG4 are not active against bacteria. Tle contains a putative lipase, and Tge has a LysM PG-binding domain and a peptidase domain, indicating that it acts on PG. Both lipase and murein hydrolase effectors have been implicated in T6SS-mediated bacterial killing (12), suggesting that Tle and Tge could be active against *E. coli*. Alternatively, Tle and Tge action may not be sufficient for a bactericidal effect. In contrast, the Δ*vgrG24* and Δ*vgrG34* mutants secreted Hcp and killed *E. coli* similarly to the wild type, which implies that effectors secreted in a VgrG2- and VgrG3-dependent manner are important for mediating bacterial killing. Indeed, mutation of *tde* or *tse* in these backgrounds, respectively, completely abrogated killing. The genetic makeup of the *vgrG2* gene cluster, including the *tde* effector and *tdi* immunity genes, is strikingly similar to that of the nuclease effector-immunity pair described in *A. tumefaciens*, suggesting that Tde functions as a nuclease (48). Supporting this is our finding that *E. coli* was rescued from Tde killing by expression of the Tdi immunity protein. Despite its clear importance for antibacterial activity, we were unable to detect any obvious toxin domains associated with Tse, and thus, this protein may represent a new family of T6SS-delivered toxins. One interesting finding is that significant amounts of *E. coli* killing by the Δ*tssM* mutant of *A. baumannii* ATCC 17978 are detected compared to *E. coli* controls. This has been described previously by us and others and suggests that additional antibacterial mechanisms exist in this strain apart from T6SS (38, 41). Furthermore, our assays showed that several combinations of *vgrG* mutants, for example, the Δ*vgrG23* mutant, were attenuated for *E. coli* killing to an even greater extent than the Δ*tssM* mutant. While the mechanistic basis of this is not known, VgrG proteins from *P. aeruginosa* and *Francisella tularensis* have been demonstrated to be secreted in a T6SS-independent manner, which may explain some of the observed T6SS-independent killing (62, 63).

Dedicated PG hydrolases are often genetically linked with macromolecular complexes that have components that cross the PG layer (37). Biochemically, these enzymes are frequently lytic transglycosylases that cleave the glycosidic linkages of the PG disaccharide backbone (64). In many cases, these hydrolases physically interact with core components of the membrane-spanning complexes, allowing them to alter the PG layer at precise cellular locations. Examples of this include VirB1 from *Brucella suis*, which interacts with other VirB T4SS components; FlgJ from *Salmonella*, which interacts with other flagellar components; and the lytic transglycosylase EtgA from *E. coli*, which is essential for T3SSs (65–68). Bacteriophage, although entering the cell from the external environment, must also possess a mechanism to cross the PG when puncturing a bacterial cell. For T4 phage, this is accomplished by the lysozyme domain of the gp5 protein (69). Interestingly, VgrG proteins show sequence and structural homology to gp5 but lack this lysozyme domain (17, 18, 30, 70, 71). Despite the requirement of a PG hydrolase for these systems, an enzyme performing this function for T6SS has yet to be described. Our genetic analysis of the *A. baumannii* ATCC 17978 and *A. baylyi* ADP1 T6SS clusters identified TagX as an essential component of the T6SS machinery, as *tagX* mutants failed to secrete Hcp. The C-terminal end of TagX shares homology with enzymatically active domains of Ply500 from *Listeria monocytogenes* phage A500 and CwlK from *Bacillus subtilis*. Both enzymes are part of the LAS family of peptidases and are l-alanoyl-d-glutamate endopeptidases that cleave the PG peptide stem (53, 54, 72). The finding that TagX and phage protein Ply500 show sequence similarity agrees with the evolutionary connection between T6SS and many bacteriophage components (17). Our experiments confirmed TagX as an inner membrane l,d-endopeptidase requiring conserved amino acid residues for activity located at its C-terminal end. Interestingly, TagX was expressed and purified as two major protein bands, but only the lower-molecular-mass protein was active, indicating that the full-length TagX protein lacks activity. As the protein was expressed with a C-terminal His tag, the N-terminal domain may possess some form of autoinhibitory activity. Autoinhibition of PG hydrolases has been previously demonstrated, and thus, TagX may be regulated in a similar fashion (73). Furthermore, the C-terminal end is highly resistant to proteinase K digestion and, although the biological relevance of this is unknown, may play a role in the regulation of TagX. It is tempting to speculate that interaction with other components of the T6SS apparatus may control the enzymatic activity of TagX, allowing for precise spatial regulation of PG degradation. TagX is present in the T6SS clusters of several other organisms, including many *Betaproteobacteria*, but is notably absent from other well-characterized T6SSs, such as those of *P. aeruginosa* and *V. cholerae*. We hypothesize that T6SS-producing organisms have evolved other mechanisms for enabling T6SS passage through the PG layer, and therefore, we predict that additional enzymes playing this role remain to be discovered.

The data presented here provide a framework and direction for future studies of the T6SS of *Acinetobacter* species. We have established the basic requirements for elaborating a functional T6SS in these organisms and demonstrated that VgrG proteins show marked differences in their relative contributions to Hcp secretion and bacterial killing. Furthermore, we have delineated the roles of a putative nuclease-immunity pair, Tde and Tdi, and an effector of unknown activity, Tse, in antibacterial activity. Our experiments suggest that these effectors require their cognate VgrG for secretion by the T6SS. Future studies on the nature of effectors secreted by this system, as well as the basis for their secretion, should further uncover the precise physiological role of the T6SSs in these organisms. Finally, our discovery of TagX presents a more complete picture of T6SS biogenesis and suggests that similar enzymes should be identifiable in other organisms.

## MATERIALS AND METHODS

### Bacterial strains and growth conditions.

For the bacterial strains and plasmids used in this study, see Table S1 in the supplemental material. Strains were grown in LB medium at 37°C with shaking. The antibiotics ampicillin (50 µg/ml), kanamycin (50 µg/ml), rifampin (150 µg/ml), and tetracycline (5 µg/ml) were added where necessary.

### RNA-seq of T6^+^ and T6^−^ cells.

Triplicate cultures of T6^+^ and T6^−^ *A. baumannii* ATCC 17978 were grown overnight, diluted into fresh medium, and grown to an optical density at 600 nm (OD_600_) of 0.5. Three hundred microliters of cells was lysed, and RNA was stabilized by using the protocols and buffers for the RNAprotect Bacteria Reagent kit (Qiagen). RNA was purified with the RNeasy minikit (Qiagen), and rRNA was depleted with the RiboZero kit (Illumina). Directional RNA-seq libraries were constructed with the PrepX RNA-Seq for Illumina library kit (Wafergen) and sequenced with an Illumina NextSeq 500 as paired-end 75-base reads. Reads were mapped as paired ends to the *A. baumannii* ATCC 17978-mff genome, raw counts and TPM values were calculated, and differentially expressed genes were identified with the DESeq2 package in R (74).

### Sample preparation for whole-cell proteomics.

Ten milligrams of freeze-dried bacterial cellular material was suspended in 4% SDS–100 mM triethylammonium bicarbonate (TEAB; pH 8.0)–20 mM dithiothreitol and boiled at 95°C at 2,000 rpm for 10 min. Dried protein pellets were resuspended in 6 M urea–2 M thiourea–40 mM NH_4_HCO_3_ and reduced/alkylated prior to digestion with Lys-C (1/200, wt/wt) and then trypsin (1/50, wt/wt) overnight as previously described (75). Digested samples were acidified to a final concentration of 0.5% formic acid and desalted with 50 mg of tC_18_ SEP-PAK (Waters Corporation, Milford, CT) according to the manufacturer’s instructions. Briefly, tC_18_ SEP-PAK was conditioned with buffer B (80% acetonitrile [ACN], 0.1% formic acid) and washed with 10 volumes of buffer A* (0.1% trifluoroacetic acid, 2% ACN), the sample was loaded, the column was washed with 10 volumes of buffer A*, and bound peptides were eluted with buffer B and then dried. For tandem mass tag (TMT) labeling, samples were resuspended in 100 mM TEAB and 100 µg of peptide according to the manufacturer’s instructions. Samples T6^+^ B1, T6^+^ B2, T6^+^ B3, T6^−^ B1, T6^−^ B2, and T6^−^ B3 were labeled with TMT labels 126 to 131, respectively. After being labeled samples were pooled and C_18_ StAGE Tips2 was used to desalt the peptides prior to fractionation by basic reverse-phase chromatography. Briefly, peptides were separated with an 1100 series HPLC instrument with a Zorbax Extend C_18_ column (1.0 by 50 mm, 3.5 µm; Agilent) at a flow rate of 100 µl/min. The following gradient was run: initial 5 min from 100% buffer A (5 mM ammonium formate, 2% acetonitrile, pH 10) to 6% buffer B (5 mM ammonium formate, 90% acetonitrile, pH 10), then in 2 min to 8% buffer B, followed by an increase to 27% buffer B in 38 min, to 31% B in 4 min, to 39% B in 4 min, to 60% B in 7 min, and completion with a 4-min run at 100% buffer B and a 26-min gradient back to 100% buffer A. Fractions of 100 µl were collected in a 96-well plate with every eighth fraction combined to generate a total of eight fractions that were concentrated by vacuum centrifugation and subjected to MS analysis.

### Liquid chromatography-tandem MS (MS/MS) analysis.

Purified peptides were resuspend in buffer A* and separated on an EASY-nLC1000 system coupled to an LTQ-Orbitrap Velos (Thermo Scientific). Briefly, samples were loaded directly onto an in-house-packed 30-cm, 75-µm-inner-diameter, 360-µm-outer-diameter Reprosil-Pur C_18_ AQ 3 µm column (Dr. Maisch, Ammerbuch-Entringen, Germany). Reverse-phase analytical separation was performed at 350 nl/min over a 180-min gradient by altering the buffer B concentration from 0 to 32% in 150 min, from 32 to 40% in the next 5 min, increasing it to 100% in 2.5 min, holding it at 100% for 2.5 min, and then dropping it to 0% for another 20 min. The LTQ-Orbitrap Velos was operated with Xcalibur v2.2 (Thermo Scientific) at a capillary temperature of 275°C with data-dependent acquisition and switching between collision-induced dissociation (CID) MS/MS (normalized collision energy [NCE], 35%; activation Q, 0.25; activation time, 10 ms; automated gain control [AGC] at 4 × 10^4^) and higher-energy collisional dissociation (HCD) MS/MS (resolution, 7,500; NCE, 45%; AGC at 2e5; maximum fill time, 200 ms).

### MS data analysis.

MS data were processed with MaxQuant (v1.5.3.28) (76). Database searching was carried out against the reference *A. baumannii* ATCC 17978 proteome (downloaded from UniProt on 16 November 2014; 3,799 proteins) and our recent resequencing genome *A. baumannii* ATCC 17978-mff proteome (42) with the following search parameters: carbamidomethylation of cysteine as a fixed modification, oxidation of methionine, acetylation of protein N-terminal trypsin/P cleavage with a maximum of two missed cleavages. TMT-6-based quantitation was enable by using the default TMT-6 settings with an MS tolerance of 6 ppm, an HCD MS/MS tolerance of 20 ppm, a CID MS/MS tolerance of 0.5 Da, and a maximum false-discovery rate of 1.0% for protein and peptide identifications. The resulting protein group output was processed within the Perseus (v1.5.0.9) analysis environment to remove reverse matches and common protein contaminants.

### Hcp secretion and bacterial killing.

Detection of Hcp in normalized samples of whole cells and culture supernatants by Western blotting was performed as described previously, with an anti-Hcp and anti-RNA polymerase antibody, which was used as a lysis control (BioLegend) (42). Except where indicated otherwise, samples were collected from cells grown for 4 h after 1:50 back dilution into fresh medium after overnight growth. Detection of Hcp in strains complemented with the VgrG1-expressing plasmids was done after overnight growth.

Bacterial killing assays were performed as follows. Cultures of *A. baumannii* and *E. coli* MG1655R were grown overnight, and the *E. coli* cells were washed three times with fresh LB to remove rifampin. The cells were then resuspended to an OD_600_ of 1.0, 100 µl of *E. coli* was mixed with 10 µl of *A. baumannii*, and a 10-µl sample was spotted onto a dry LB agar plate. After a 4-h incubation at 37°C, spots were cut from the agar and resuspended by mixing with 500 µl of LB broth. This mixture was serially diluted 10-fold, and dilutions were plated onto rifampin-containing LB agar to determine the number of *E. coli* CFU remaining. Controls consisted of *E. coli* MG1655R mixed with rifampin-sensitive *E. coli* TOP10 or LB medium. Experiments were performed twice in technical triplicate. For analysis of Tsi2, experiments were performed as described above but with a tetracycline-resistant *E. coli* strain either containing a pEXT20 control vector or expressing pTdi. Competitions for these experiments were done at a 1:20 *A. baumannii*-to-*E. coli* ratio, and *E. coli* bacteria were enumerated on agar plates containing tetracycline.

### Construction of *A. baumannii* mutants, cloning, and Western blotting.

For the primers used in this study, see Table S2 in the supplemental material. Mutants were constructed as described previously (50). Briefly, an antibiotic resistance cassette was amplified with ~110-bp oligonucleotide primers (Integrated DNA Technologies) with homology to the flanking regions of the targeted gene with an additional 3′ 18 to 25 nucleotides of homology to the FRT site-flanked kanamycin resistance cassette from plasmid pKD4. This PCR product was electroporated into competent T6^+^ *A. baumannii* ATCC 17978 carrying pAT04, which expresses The Rec_Ab_ recombinase. Mutants were selected on 7.5 µg/ml kanamycin, and integration of the resistance marker was confirmed by PCR. To remove the kanamycin resistance cassette, electrocompetent mutants were transformed with pAT03, which expresses the FLP recombinase, to remove the FRT-flanked resistance cassette. Additional mutations were made by repeating this process, and PCR and sequencing were used to confirm the validity of all of the mutants.

The *vgrG1* gene from *A. baumannii* ATCC 17978 was cloned with a 10-His tag into the BamHI and PstI sites of pBAVMCS with primers vgrG1FwdBamHI and vgrG1RevPstI10His. The *tagX* gene from *A. baylyi* was cloned into the PacI and NotI sites of the pEVL174 vector (43) with primers ACIAD2699-pacI and ACIAD2699-notI, and the *tagX* gene from *A. baumannii* ATCC 17978 was cloned into the BamHI and SalI sites of pEXT20 with primers tagXFwdBamHI and tagXFwdSalI. This construct was used as a template for point mutations. Cloning of the *A. baumannii* tagX gene into expression vector pBAVMCS was performed with primers tagXpromFwdPstI and tagXRevPstI6His with the pTagX vector used as a template in order to maintain the pEXT20 promoter for constitutive expression. The *tdi* gene was cloned into the KpnI/SalI sites of pEXT20 with a C-terminal FLAG tag with primers tdiFwd and tdiRev. Expression of proteins was confirmed by Western blotting with rabbit polyclonal anti-His (Pierce) and anti-FLAG (Sigma) antibodies. Detection of primary antibodies was done with fluorescent secondary antibodies as previously described (38).

### Point mutations.

The QuikChange site-directed mutagenesis strategy was utilized to make the TagX point mutants. Briefly, the pEXT20 plasmid containing the 6×His-tagged version of TagX was used as the template for the PCR. Complementary oligonucleotides containing the desired mutation were used as primers, and *Pfu* turbo was utilized for the extension reaction. After completion of the PCR, the products were digested with DpnI to degrade the template DNA and then transformed into TOP10 cells. Sequencing was used to confirm mutations.

### Purification of TagX and point mutant TagX proteins.

Overnight cultures of BL21 cells bearing the pEXT20 plasmid with TagX containing a C-terminal 6×His tag were back diluted 1:100 into 1 liter of Luria broth and grown at 37°C to an OD_600_ of ~0.6. The cultures were induced with 1 mM isopropyl-β-d-thiogalactopyranoside for 3 h, and the cells were collected by centrifugation at 5,000 × *g* for 10 min at 4ºC. The cells were resuspended in purification buffer (25 mM Tris [pH 7.5], 200 mM NaCl, 10% glycerol) and lysed, and cell debris was removed by low-speed centrifugation. The supernatant was collected, and membranes were isolated by ultracentrifugation at 100,000 × *g* for 1 h. The supernatant was removed, and membranes were solubilized in resuspension buffer (purification buffer plus 2% Triton X-100) to extract TagX from the membrane. The solution was ultracentrifuged again to remove the extracted membranes, and the supernatant was loaded onto an Ni-nitrilotriacetic acid agarose column, washed with purification buffer and buffer supplemented with 10 and 40 mM imidazole, and eluted with buffer supplemented with 300 mM imidazole.

### Purification of PG and zymogram assays.

PG was isolated from *A. baumannii* ATCC 17978 as described by Hoyle and Beveridge (77). After inoculation into 1-liter cultures and growth overnight, cells were pelleted, resuspended in water at a density of 200 g/liter, and then added dropwise to an equal volume of boiling 8% SDS. Once all of the cell suspension was added, the mixture was boiled for 3 h and then ultracentrifuged at 100,000 × *g* for 1 h at room temperature to pellet the PG. The supernatant was removed, and the pellet was washed five times with deionized water to remove residual SDS. The washed PG was lyophilized overnight until dry and then weighed to determine the yield. Purified TagX samples resuspended in 2× Laemmli buffer were separated by 12% bis-Tris SDS-PAGE containing 0.1% (wt/vol) PG and then washed with water for 1 h at room temperature with multiple changes of the water in order to remove residual SDS. The gel was then equilibrated in renaturation buffer (10 mM Tris-HCl [pH 7], 0.1% Triton X-100, 10 mM ZnCl_2_) for 1 h; this was followed by the addition of fresh buffer and incubation at 37°C with agitation. Water was used to wash the gel, methylene blue stain (0.1% methylene blue, 0.01% KOH) was added, and the gel was incubated for 1 h. Water washes were used to destain the gel until the bands of degraded PG were visible.

### Limited proteolysis of TagX.

Samples of purified TagX in purification buffer at a concentration of 15 µM were digested with proteinase K (200-µg/ml final concentration) on ice. The reaction was quenched after 0.25, 0.5, 0.75, 1, 2.5, 5, 10, 15, 20, 30, 40, 50, or 60 min by adding 5 mM phenylmethylsulfonyl fluoride (PMSF) and 1× SDS-PAGE loading buffer. A control sample was added to 5 mM PMSF and 1× SDS-PAGE buffer before the addition of proteinase K. Once the digestion was complete, the samples were analyzed by Western blot and zymogram assays as described above.

### Cellular localization of TagX.

Overnight cultures of *A. baumannii* ATCC 17978 cells expressing TagX from pBAVMCS were diluted 1:100 and grown until an OD_600_ of 0.6 was reached. The cells were then pelleted, and cell fractionation was performed as described by Luckett et al. (78), with minor modifications. Briefly, the cells were washed three times, resuspended in 25 mM Tris (pH 7.4)—1 mM EDTA—20% sucrose, and then incubated for 10 min. Lysozyme was added at a final concentration of 1 mg/ml to the sample used for membrane fractions to increase the efficiency of cell lysis. After centrifugation, the pellet was resuspended in 0.5 mM magnesium sulfate and incubated on ice for 10 min. For soluble fractionation, the sample was centrifuged to obtain the periplasmic fraction (supernatant). The pellet was resuspended in 25 mM Tris (pH 7.4) and freeze-thawed, magnesium chloride and DNase were added, and the mixture was incubated at 37°C for 15 min. Centrifugation was performed, and the supernatant, containing the cytoplasmic fraction, was collected. To obtain the membrane fractions, the sample was freeze-thawed and then centrifuged to remove unlysed cells. The supernatant was transferred to a fresh tube and centrifuged at 150,000 × *g* to isolate total membranes. The pellet was resuspended in 0.7% sodium lauryl sarcosine to extract inner membrane proteins. After ultracentrifugation, the supernatant contained the inner membrane fraction and the pellet, containing the outer membrane fraction, was resuspended in 25 mM Tris (pH 7.4). Trichloroacetic acid precipitation of all samples was performed, followed by SDS-PAGE and Western blot analysis by probing with antibodies to RNA polymerase, the 6×His tag (TagX and PglC), and OmpA (79).

### Analysis of muropeptides.

Purified PG or PG digested with TagX was suspended in 10 ml of 10 mM Tris-HCl (pH 7.2) and digested with 100 mg/liter α-amylase (Sigma-Aldrich, St. Louis, MO) for 1 h at 37°C. This was followed by digestion with 100 mg/liter preactivated pronase E (Merck, Darmstadt, Germany) at 60°C for 90 min. Amylase and pronase were inactivated by boiling for 20 min in 1% SDS, and the PG was collected and washed as described above for purification. The collected PG was digested with 100 g/ml cellosyl muramidase (Hoechst AG, Frankfurt, Germany) in 50 mM phosphate buffer (pH 4.9) at 37°C for 16 h. The enzyme was inactivated by being boiled for 10 min in a water bath and then centrifuged at 14,000 rpm for 5 min to remove insoluble debris. The supernatant was mixed with 1/5 volume of 0.5 M sodium borate buffer (pH 9.0) and reduced with excess sodium borohydride (NaBH_4_) for 30 min at room temperature. The pH was measured with indicator strips (Acilit; Merck) and adjusted to 3.0 with orthophosphoric acid. The samples were filtered (Millex-GV filters, 0.22-µm pore size, 2.5-mm diameter; Millipore, Cork, Ireland) and injected into the HPLC column. Separations were performed on a Breeze 2 HPLC system consisting of a 1525 binary HPLC pump (model code 5CH; Waters), a 2489 UV-visible light detector (Waters), a model 7725i manual injector (Rheodyne), and an Aeris Peptide XB-C_18_, 3.6 µm, 250- by 4.6-mm reverse-phase column (Phenomenex). The column was equilibrated at 45°C, and separation of individual muropeptides (detection wavelength of 204 nm) was performed in a linear gradient. The mobile-phase (A, 50 mM sodium phosphate [pH 4.35]; B, 75 mM sodium phosphate, 15% methanol [pH 4.95]) gradient consisted of elution at 1.0 ml/min with 100% A for 5 min, followed by a 60-min linear gradient to 0% A–100% B and then 100% B for 5 min.

Molecular mass analysis of isolated muropeptides by MS with a model LTQ-Velos linear ion trap from Thermo-Scientific was carried out in the CBMSO Protein Chemistry Facility, which belongs to the ProteoRed network, PRB2-ISCIII.

### Accession number(s).

All of the RNA-seq data obtained in this study are available in the NCBI Sequence Read Archive under the BioProject identifier PRJNA288605. RNA-seq data were deposited in the NCBI Short Read Archive and assigned accession numbers SRR3017659, SRR3017662, SRR3017665, SRR3017667, SRR3017669, and SRR3017671.

## SUPPLEMENTAL MATERIAL

Figure S1  Complementation of Δ*vgrG1* mutation. Whole-cell pellets (P) and supernatants (S) from samples grown overnight were separated by SDS-PAGE and probed with anti-His and anti-RNA polymerase antibodies. Download Figure S1, TIF file, 0.5 MB

Figure S2  Expression of Tdi protects *E. coli* from killing by T6^+^ Δ*vgrG34*. Competition assays with *E. coli* prey strains either containing the empty vector (pEXT20) or expressing the putative Tsi2 immunity protein (pTdi). The predator *A. baumannii* strains are shown on the *x* axis. The results shown are from three independent experiments, performed in duplicate. Asterisks indicate statistical significance (unpaired, two-tailed Student *t* test; **, *P* < 0.01). Download Figure S2, TIF file, 0.2 MB

Figure S3  Bioinformatic analysis of TagX. (A) Amino acid sequence of TagX, with predicted transmembrane domains highlighted. (B) Graphical view of the predicted transmembrane domains and orientation of TagX, showing C-terminal position in periplasm, with the online tool TMHMM (81). (C, D) Hydrophobicity (http://web.expasy.org/protscale/) (C) and charge (http://www.bioinformatics.nl/cgi-bin/emboss/charge) (D) plots of TagX showing positive charges located after the first transmembrane segment. Download Figure S3, TIF file, 2.6 MB

Figure S4  Purification of TagX and point mutant protein TagX^D287N^. Coomassie gel of flowthrough, wash, and elution fractions from the purifications. Download Figure S4, TIF file, 0.9 MB

Table S1  Strains and plasmids used in this study. Table S1, DOCX file, 0.02 MB

Table S2  Primers used in this study. Table S2, DOCX file, 0.01 MB
