# Supplementary figures and images for: Genetic Dissection of the Type VI Secretion System in Acinetobacter and Identification of a Novel Peptidoglycan Hydrolase, TagX, Required for Its Biogenesis
Source: mBio. 2016 Oct 11;7(5):e01253-16. doi: 10.1128/mBio.01253-16 (PMC5061870; doi:10.1128/mBio.01253-16)

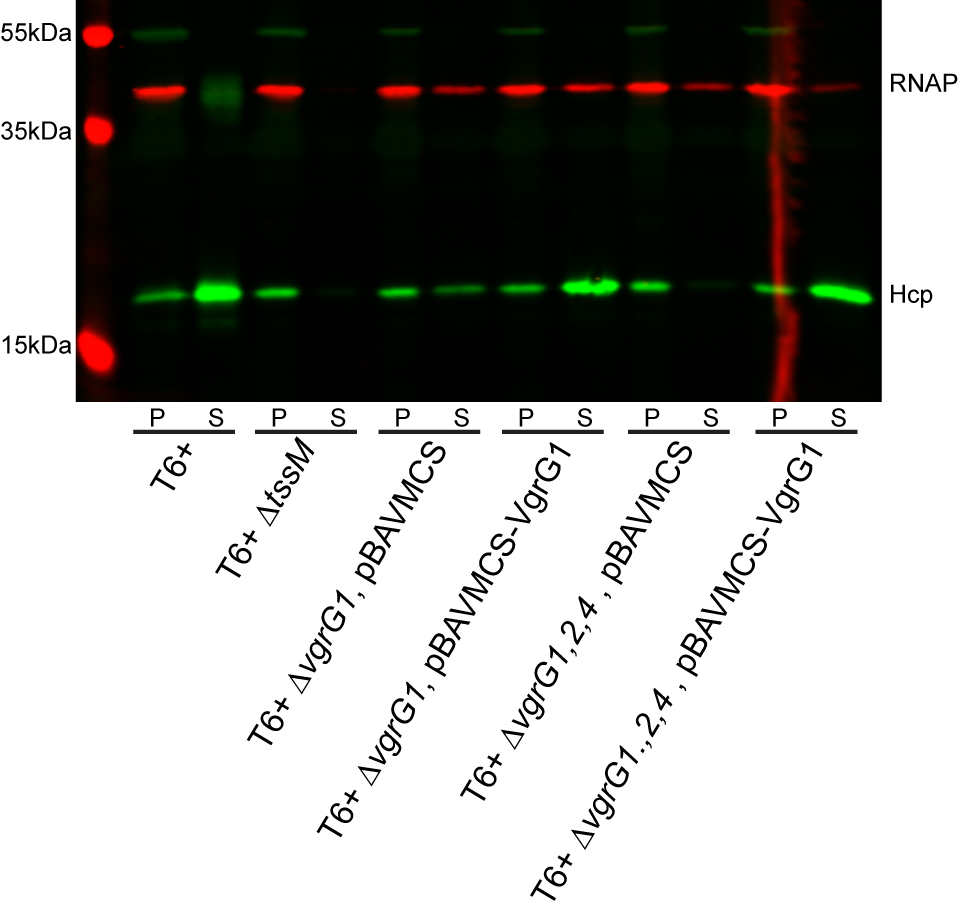

Supplement: Figure S1 — Complementation of ΔvgrG1 mutation. Whole-cell pellets (P) and supernatants (S) from samples grown overnight were separated by SDS-PAGE and probed with anti-His and anti-RNA polymerase antibodies. Download [file mbo005163027sf1.tif]

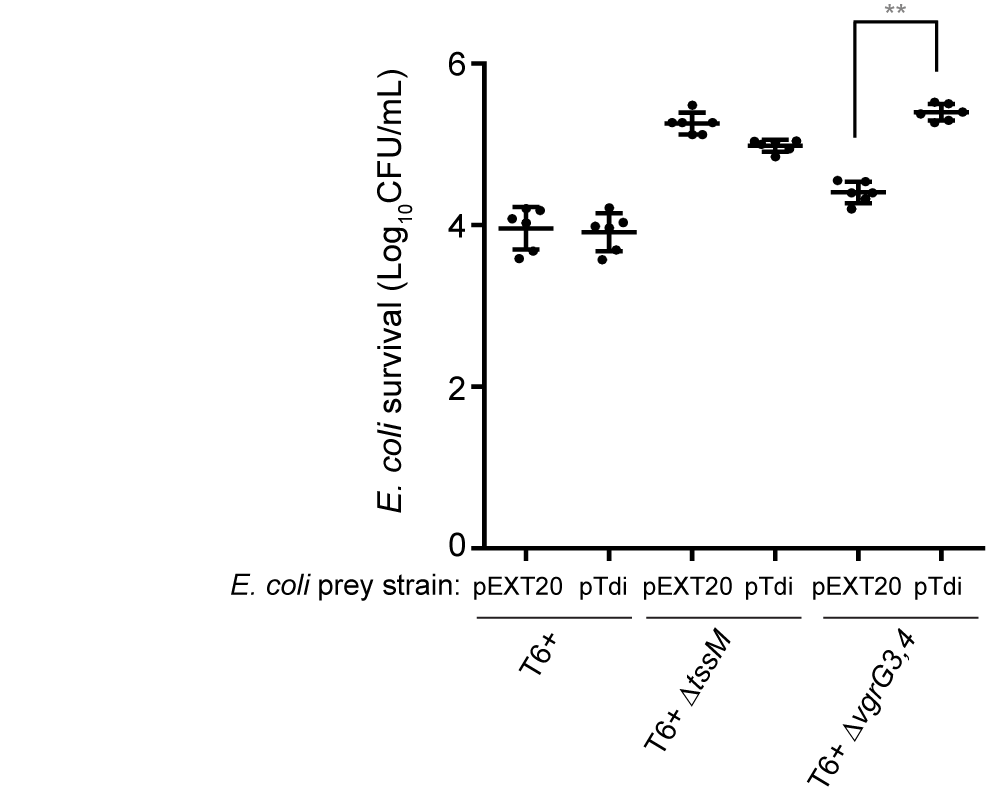

Supplement: Figure S2 — Expression of Tdi protects E. coli from killing by T6+ ΔvgrG34. Competition assays with E. coli prey strains either containing the empty vector (pEXT20) or expressing the putative Tsi2 immunity protein (pTdi). The predator A. baumannii strains are shown on the x axis. The results shown are from three independent experiments, performed in duplicate. Asterisks indicate statistical significance (unpaired, two-tailed Student t test; **, P < 0.01). Download [file mbo005163027sf2.tif]

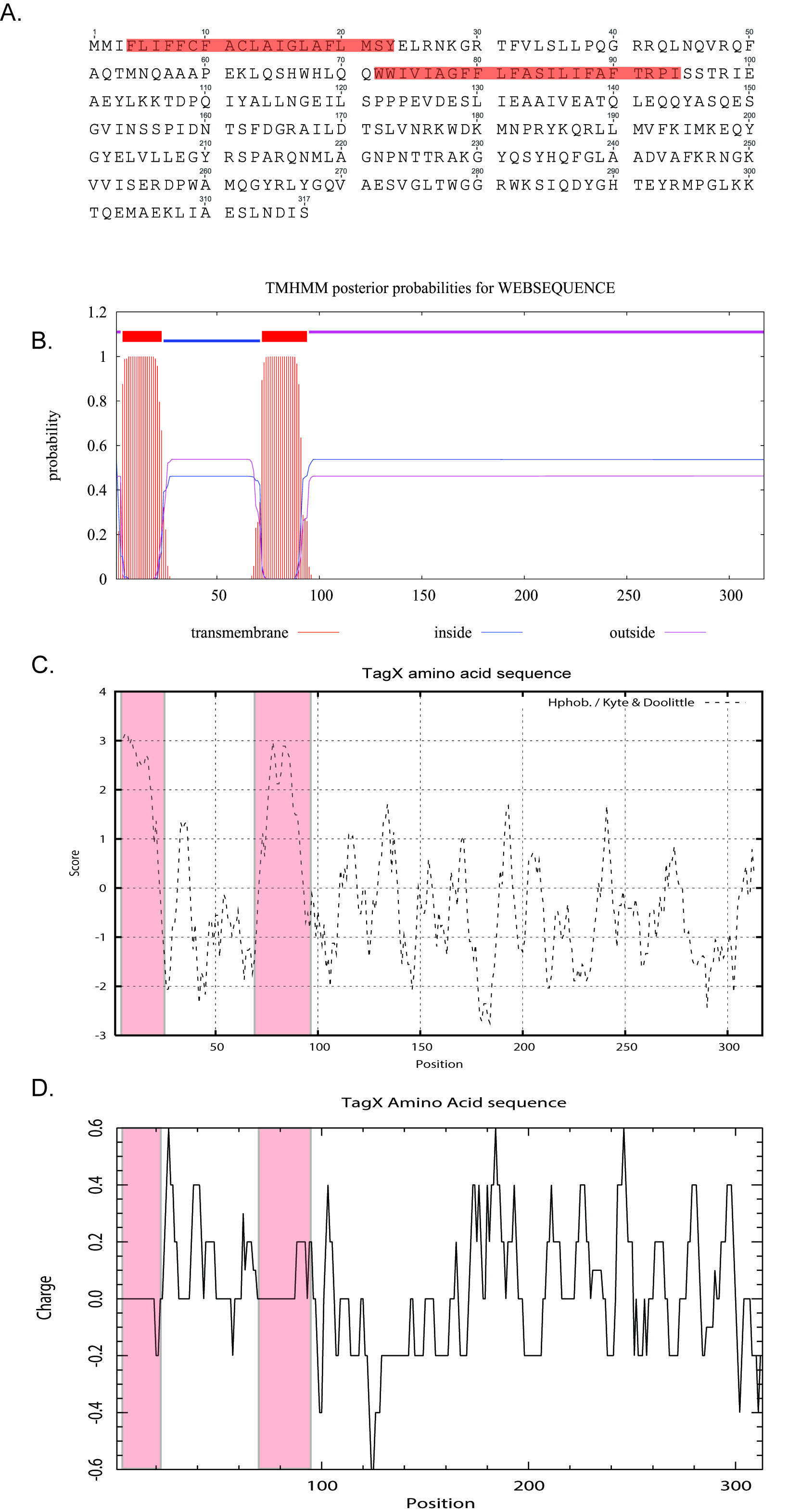

Supplement: Figure S3 — Bioinformatic analysis of TagX. (A) Amino acid sequence of TagX, with predicted transmembrane domains highlighted. (B) Graphical view of the predicted transmembrane domains and orientation of TagX, showing C-terminal position in periplasm, with the online tool TMHMM (81). (C, D) Hydrophobicity (http://web.expasy.org/protscale/) (C) and charge (http://www.bioinformatics.nl/cgi-bin/emboss/charge) (D) plots of TagX showing positive charges located after the first transmembrane segment. Download [file mbo005163027sf3.tif]

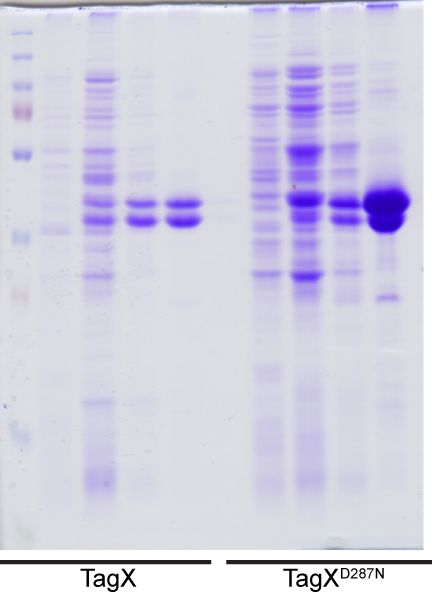

Supplement: Figure S4 — Purification of TagX and point mutant protein TagXD287N. Coomassie gel of flowthrough, wash, and elution fractions from the purifications. Download [file mbo005163027sf4.tif]
