# Supplementary material for: Genetic Dissection of the Type VI Secretion System in Acinetobacter and Identification of a Novel Peptidoglycan Hydrolase, TagX, Required for Its Biogenesis
Source: mBio. 2016 Oct 11;7(5):e01253-16. doi: 10.1128/mBio.01253-16 (PMC5061870; doi:10.1128/mBio.01253-16)
Supplement: Table S1 — Strains and plasmids used in this study. [file mbo005163027st1.docx]

| Strain or plasmid | Relevant characteristics | Reference |
| --- | --- | --- |
| *A. baylyi* ADP1 | wild type strain | (43) |
| *A. baylyi* ADP1 ∆ACIAD2678 | mutant strain |  |
| *A. baylyi* ADP1 ∆ACIAD2680 | mutant strain | (43) |
| *A. baylyi* ADP1 ∆ACIAD2681 | mutant strain | (43) |
| *A. baylyi* ADP1 ∆ACIAD2682 | mutant strain | (43) |
| *A. baylyi* ADP1 ∆ACIAD2683 | mutant strain | (43) |
| *A. baylyi* ADP1 ∆ACIAD2684 | mutant strain | (43) |
| *A. baylyi* ADP1 ∆ACIAD2685 | mutant strain | (43) |
| *A. baylyi* ADP1 ∆ACIAD2686 | mutant strain | (43) |
| *A. baylyi* ADP1 ∆ACIAD2687 | mutant strain | (43) |
| *A. baylyi* ADP1 ∆ACIAD2688 | mutant strain | (43) |
| *A. baylyi* ADP1 ∆ACIAD2689 | mutant strain | (43) |
| *A. baylyi* ADP1 ∆ACIAD2690 | mutant strain | (43) |
| *A. baylyi* ADP1 ∆ACIAD2691 | mutant strain | (43) |
| *A. baylyi* ADP1 ∆ACIAD2693 | mutant strain | (43) |
| *A. baylyi* ADP1 ∆ACIAD2694 | mutant strain | (43) |
| *A. baylyi* ADP1 ∆ACIAD2695 | mutant strain | (43) |
| *A. baylyi* ADP1 ∆ACIAD2696 | mutant strain | (43) |
| *A. baylyi* ADP1 ∆ACIAD2697 | mutant strain | (43) |
| *A. baylyi* ADP1 ∆ACIAD2698 | mutant strain | (43) |
| *A. baylyi* ADP1 ∆ACIAD2699 | mutant strain (*tagX*) | (43) |
| *A. baylyi* ADP1 ∆ACIAD2700 | mutant strain | (43) |
| *A. baylyi* ADP1 ∆ACIAD2704 | mutant strain | (43) |
| *A. baylyi* ADP1 ∆ACIAD2708 | mutant strain | (43) |
| *A. baylyi* ADP1 ∆ACIAD2716 | mutant strain | (43) |
| *A. baylyi* ADP1 ∆ACIAD2699 pEVL17-TagX | mutant strain, expressing *A. baylyi* TagX (ACIAD2699) | This study |
| *A. baumannii* ATCC17978 T6+ | T6SS active strain | (40) |
| *A. baumannii* ATCC17978 T6- | T6SS inactive strain | (40) |
| *A. baumannii* ATCC17978 T6+ ∆*tssM* | mutant strain | (40) |
| *A. baumannii* ATCC17978 T6+ ∆*vgrG1* | mutant strain | This study |
| *A. baumannii* ATCC17978 T6+ ∆*vgrG2* | mutant strain | This study |
| *A. baumannii* ATCC17978 T6+ ∆*vgrG3* | mutant strain | This study |
| *A. baumannii* ATCC17978 T6+ ∆*vgrG4* | mutant strain | This study |
| *A. baumannii* ATCC17978 T6+ ∆*vgrG1*,*2* | mutant strain | This study |
| *A. baumannii* ATCC17978 T6+ ∆*vgrG1*,*3* | mutant strain | This study |
| *A. baumannii* ATCC17978 T6+ ∆*vgrG1*,*4* | mutant strain | This study |
| *A. baumannii* ATCC17978 T6+ ∆*vgrG2*,*3* | mutant strain | This study |
| *A. baumannii* ATCC17978 T6+ ∆*vgrG2*,*4* | mutant strain | This study |
| *A. baumannii* ATCC17978 T6+ ∆*vgrG3,4* | mutant strain | This study |
| *A. baumannii* ATCC17978 T6+ ∆*vgrG1*,*2,3* | mutant strain | This study |
| *A. baumannii* ATCC17978 T6+ ∆*vgrG1,2,4* | mutant strain | This study |
| *A. baumannii* ATCC17978 T6+ ∆*vgrG1,3,4* | mutant strain | This study |
| *A. baumannii* ATCC17978 T6+ ∆*vgrG2*,*3*,*4* | mutant strain | This study |
| *A. baumannii* ATCC17978 T6+ ∆*vgrG1*,*2*,*3*,*4* | mutant strain | This study |
| *A. baumannii* ATCC17978 T6+ ∆*vgrG2*,*3*,*4*∆*tse3* | mutant strain | This study |
| *A. baumannii* ATCC17978 T6+ ∆*vgrG1,* pBAVMCS | mutant strain, vector control | This study |
| *A. baumannii* ATCC17978 T6+ ∆*vgrG1,* pBAVMCS-VgrG1 | mutant strain, expressing VgrG1 | This study |
| *A. baumannii* ATCC17978 T6+ ∆*vgrG1*,*2*,*4,* pBAVMCS | mutant strain, vector control | This study |
| *A. baumannii* ATCC17978 T6+ ∆*vgrG1*,*2*,*4,* pBAVMCS-VgrG1 | mutant strain, expressing VgrG1 | This study |
| *A. baumannii* ATCC17978 T6+ ∆*tagX* | mutant strain | This study |
| *A. baumannii* ATCC17978 T6+ ∆*tagX* pBAVMCS | mutant strain with vector, Kan^R^ | This study |
| *A. baumannii* ATCC17978 T6+ ∆*tagX* pBAVMCS-TagX | mutant strain expressing TagX, Kan^R^ | This study |
| *E. coli* MG1655R | *E. coli* strain used for killing assays, Rif^R^ | (67) |
| pBAVMCS | cloning vector, Kan^R^ | (69) |
| pBAVMCS-VgrG1 | Construct expressing 10His-tagged VgrG1 | This study |
| pEXT20 | Cloning Vector, Amp^R^, IPTG inducible | (68) |
| pTdi | pEXT20 construct expressing *A. baumannii* Tdi, Amp^R^, IPTG inducible | This study |
| pTagX | pEXT20 construct expressing *A. baumannii* TagX, Amp^R^, IPTG inducible | This study |
| pTagX^D287N^ | pEXT20 construct expressing *A. baumannii* TagX mutant, Amp^R^, IPTG inducible | This study |
| pBAVMCS-TagX | Construct expressing *A. baumannii* 6His-tagged TagX with pEXT20 promoter, Kan^R^ | This study |
| pEVL17-TagX | Construct expressing *A. baylyi* TagX, Amp^R^ | This study |
|  |  |  |
