# Supplementary material for: Genetic Dissection of the Type VI Secretion System in Acinetobacter and Identification of a Novel Peptidoglycan Hydrolase, TagX, Required for Its Biogenesis
Source: mBio. 2016 Oct 11;7(5):e01253-16. doi: 10.1128/mBio.01253-16 (PMC5061870; doi:10.1128/mBio.01253-16)
Supplement: Table S2 — Primers used in this study. [file mbo005163027st2.docx]

| **Primer name** | **sequence (5’-3’)** | **Purpose** |
| --- | --- | --- |
| tagXFwdBamHI | ATA TGG ATC CAT GAT GAT TTT TCT AAT TTT CTT TTG TTT TGC | clone tagX into pEXT20 with 6-His tag |
| tagXRevSalI6His | ATA GTC GAC TTA GTG GTG ATG GTG ATG ATG ACT GAT GTC ATT TAA GCT TTC GGC | clone tagX into pEXT20 with 6-His tag |
| tagXpromFwdPstI | ATA CTG CAG TTG CGC CGA CAT CAT AAC GGT TCT GG | clong tagX into pBAVMCS with pEXT20 promoter, 6-His tag |
| tagXRevPstI6His | ATA CTG CAG TTA GTG GTG ATG GTG ATG ATG ACT GAT GTC ATT TAA GCT TTC GGC | clong tagX into pBAVMCS with pEXT20 promoter, 6-His tag |
| ACIAD2699-pac | CCCTTAATTAATGTTTAAAGCGCTACTTCCTCAGAGTAAACAAAAAC | clone *A. baylyi tagX* into pEVL17 |
| ACIAD2699-not | TTTGCGGCCGCTTAACTGGCTGCATTGGCAGTTAATTGG | clone *A. baylyi tagX* into pEVL17 |
| vgrG1FwdBamHI | ATA GGA TCC ATG CAG ATG AGT GTG TCG AGT ATA TTG G | clone vgrG1 into pBAVMCS  with 10-His tag |
| vgrG1RevPstI10His | ATA CTG CAG TCA GTG GTG ATG ATG GTG GTG ATG GTG ATG ATG ATT TAC CAC ATG TAT TTT ATA TTG C | clone vgrG1 into pBAVMCS  with 10-His tag |
| vgrG1KOFwd | TTA CTG GCG GCA CAT TAC TTA CTT TTC CAT GTG CTT TTT TTA ACA ATT CAG TCG CTT GTT TTT TTA TAT CTT TAT TGG CAT CTA TTA CAT TAT CCA TTT CAA TTA GCG ATT GTG TAG GCT GGA GCT GCT TCG | mutate vgrG1 |
| vgrG1KORev | TTA GAC TAG TTA CAT TTT GAA AAT GAG TAT AGA TAG ACA GAT TAG TCT TTT TTT AAT TAA AAT ATG GCT TAA ATA TAA GTT TGA TTG ATA ATA AAA ATG CAG CAT ATG AAT ATC CTC CTT AGT TCC TAT TCC G | mutate vgrG1 |
| vgrG2KOFwd | TTT CAG CGC CAT ACC AAA ACT GCT GTT GTA TTT CCG TAG GTC GTT CTG CGA TCT TTG ACC AGA ATT GTT GAT TTT GAG GAT CTG GCA TAA TTC TTT ATT CAG CGA TTG TGT AGG CTG GAG CTG CTT CG | mutate vgrG2 |
| vgrG2KORev | TAG CCA TTA ACA TTG CAA TTG ATA TAA AAA ATA GAC AGA TCT GTA CGC ATT TTA TAA AAT ATA ATC GAT TAA GTC TCA ATT TAT AAC AAA CGT AAT GTT TCA TAT GAA TAT CCT CCT TAG TTC CTA TTC CG | mutate vgrG2 |
| vgrG3KOFwd | AAG ATT ATA CTA TAT TTT ACA TTT GGT AAT TCT TTA TTT CTT TGA TTA TAA AAG ACT ATT GTA TAT TCA AAA ATA GTA TAA GGA TTC ATT ATA TCT TTA ACC AGC GAT TGT GTA GGC TGG AGC TGC TTC G | mutate vgrG3 |
| vgrG3KORev | TAA GTT AGG GGA AGA TAA GCT AAT TTA TAT AAA TTA GAC AAT TCT GTC TGT TTT TTA TAA AAT ATC GAT GAA TAT GAC TCA GTT TTT ATA ATA ATG ATG CAT ATG AAT ATC CTC CTT AGT TCC TAT TCC G | mutate vgrG3 |
| vgrG4KOFwd | AAA TTG ATA GAA ATA ACG CAT AAT TTT GTA GAC AGA TAG TTC TGT TTT TTA TAA AGT GCA GCC GTT TAT ATA TCC TTA TGA TTA TAA AAA AAT GCT TAG CGA TTG TGT AGG CTG GAG CTG CTT CG | mutate vgrG4 |
| vgrG4KORev | ATA AGA TTA ACC CGT TAG GTC AGG TGG CCT AAC TTA AAT AAA AAA GTC TCC GAC AAA CTC GGT ACG GTT CAA TTT ATA ACT ATC ATT TAT TTT TTC ATA CAT ATG AAT ATC CTC CTT AGT TCC TAT TCC G | mutate vgrG4 |
| tse2KOFwd | ATG ATG TCC TCT GTC ACC TCA ACT TTA TCA ATT GGT TGA CCA TAT TTC TCA TAA AAG TAA TCA ATA TCT TCT TGG TTC ATC TTA CTA CCC TCT ATT ACT TAG CGA TTG TGT AGG CTG GAG CTG CTT CG | mutate tse2 |
| tse2KORev | AAG ACC AAA GTA AAG ATG AAT TTC CAG CTT TAG AAG TAG ATG ACT GGT TTA CTC AAC TTG GAA GCA GTA CAA AGA CTG GAA AAG AGG AAT AAA GAA TTA TGC CAC ATA TGA ATA TCC TCC TTA GTT CCT ATT CCG | mutate tse2 |
| tse3KOFwd | TTA ACA AAA AAA CAT CAT CTT GAC TTA AAC CAA AAT AAT TCT GTC AGA TGT GCG GTT AAT TAA ACC AAA TCC TAT AGA AAC ATA TAA TAT AAT TAC ACA GCG ATT GTG TAG GCT GGA GCT GCT TCG | mutate tse3 |
| tse3KORev | AAG ATT TGA AAC TGA TGG CTC AGA ACA AAT TTC CAT TCA TAT TTG TGA TGA AAA TGC TTC TAA ATA TAA ACT AGC GGC AAA AGG TTA AAG ATA TAA TGA ATC ATA TGA ATA TCC TCC TTA GTT CCT ATT CCG | mutate tse3 |
| tagXKOFwd | TCC GGT TGA ACA AGA GGC TGA TGT GGA ACC ACC TAA AGC TTC GGT TGA TAT TAA GAT TAC ACG AAG TCC ATT TGC TGT GAA TTA GTT AAG TCA TGA TGA TTA GCG ATT GTG TAG GCT GGA GCT GCT TCG | mutate tagX |
| tagXKORev | AAT TTA TAC ATG GGA AAA AGC TGG GTT GAT TTC CCA GCT TGT CGA TCA CAT GAC TAC CGT TTT ACA TGA TCG CTT CCA ATC AGG CTC CTA GGT TTA ACT CAT ATG AAT ATC CTC CTT AGT TCC TAT TCC G | mutate tagX |
| tagX^D287N^ | GCAAAAGGGTATCAAAGTTATTTTCAATTTGGTTTAGCGGCAAATGTGGCCTTTAAGCG | point mutant of tagX |
| tdiFwd | ATT GGT ACC ATG AAC CAA GAA GAT ATT GAT TAC | cloning tdi |
| tdiRev | ATG TCG ACT CAC TTG TCG TCA TCG TCT TTG TAG TCG TTC TCA CCA TCC CAA TAC CAA TAC | cloning tdi |
